# From Mechanism to Medicine: Peptide-Based Approaches for Cancer Diagnosis and Therapy

**DOI:** 10.3390/biom16010027

**Published:** 2025-12-24

**Authors:** Maria João Gouveia, Joana Campanhã, Francisca Barbosa, Nuno Vale

**Affiliations:** 1 PerMed Research Group, RISE-Health, Department of Community Medicine, Health Information and Decision (MEDCIDS), Faculty of Medicine, University of Porto, Alameda Professor Hernâni Monteiro, 4200-319 Porto, Portugal; mariajoaogouveia@gmail.com (M.J.G.); joanacsantos2002@gmail.com (J.C.); francisca.obc@hotmail.com (F.B.); 2 Faculty of Pharmacy, University of Porto, Rua de Jorge Viterbo Ferreira, 228, 4050-313 Porto, Portugal; 3 Laboratory of Personalized Medicine, Department of Community Medicine, Health Information and Decision (MEDCIDS), Faculty of Medicine, University of Porto, Rua Doutor Plácido da Costa, 4200-450 Porto, Portugal; 4 RISE-Health, Department of Community Medicine, Health Information and Decision (MEDCIDS), Faculty of Medicine, University of Porto, Rua Doutor Plácido da Costa, 4200-450 Porto, Portugal

**Keywords:** therapeutic peptides, targeted drug delivery, peptide–drug conjugates, peptide-guided radionuclides, peptide-based vaccines, protein–protein interaction inhibitors, cancer immunotherapy, precision oncology, extracellular vesicles, engineered cells

## Abstract

Therapeutic peptides have rapidly evolved into multifunctional tools for precision oncology, offering molecular specificity and biocompatibility. Their roles in cancer therapy, however, are inherently overlapping. The same peptide can function as a targeting ligand, a cell-penetrating motif, a therapeutic effector, or a structural component of peptide–drug conjugates (PDCs), nanoparticle (NP) systems, and radionuclide constructs. This functional convergence makes rigid classification challenging. In this review, we therefore organize peptide modalities according to their dominant therapeutic function while acknowledging the fluid boundaries between categories. Firstly, we outline the main functional classes of therapeutic peptides, covering their use as targeting ligands and their roles as active agents (i.e., receptor agonists/antagonists, intracellular protein–protein interaction modulators, etc.). Additionally, we summarize their application in peptide–drug conjugates (PDCs), peptide-guided radionuclides, and cancer vaccines, integrating key mechanistic principles and clinical evidence. Finally, we discuss the major translational barriers to clinical use and how they might be overcome. The developments in peptide engineering position them as adaptable, multifunctional platforms capable of improving precision, reducing toxicity, and advancing personalized cancer care.

## 1. Introduction

Cancer remains one of the leading causes of morbidity and mortality worldwide, with lung cancer representing the main cause of cancer-related deaths in men and breast cancer (BC) in women [1,2,3]. Conventional therapies, including chemotherapy, radiotherapy, hormone therapy, and surgery, have significantly improved patient survival and quality of life. Nevertheless, these approaches are frequently associated with severe adverse effects, such as oxidative stress, DNA damage, immunosuppression, and off-target toxicity to healthy tissues, as well as complications related to surgery, infections, and drug resistance [3]. These limitations underscore the urgent need for safer and more precise therapeutic options [4].

Therapeutic peptides have emerged as promising alternatives due to their high specificity, biocompatibility, and versatility [5]. Unlike conventional therapies, peptides are biodegradable, exhibit lower immunogenicity, and are less prone to tissue accumulation, translating into improved safety profiles. Advances in peptide synthesis and biotechnological production have further expanded their applicability, making them accessible for large-scale clinical use [5,6]. Therapeutic peptides have already shown versatility across biomedical fields, from infectious and metabolic diseases to neurodegenerative disorders and tissue regeneration. In oncology, peptides offer dual functionality. First, they can serve as targeting ligands for precision delivery platforms, including nanoparticles, exosomes, or engineered cells, thereby enhancing tumor selectivity and reducing systemic toxicity. Second, they can act as functional therapeutics, including cytotoxic agents, receptor antagonists, intracellular protein–protein interaction modulators, or immune modulators within anticancer vaccines and immunotherapies [7,8,9].

This review provides a comprehensive analysis of the diverse classes of therapeutic peptides in cancer, highlighting their mechanisms of action, innovative delivery strategies, and translational potential. We focus on peptide conjugate modalities, functional therapeutic peptides, and peptide-based immune modulation and discuss current clinical advances and challenges in translating peptides from bench to bedside.

## 2. Peptide Conjugate Modalities for Target Therapy

Peptides have evolved from bioactive molecules with intrinsic anticancer activity into versatile targeting ligands capable of directing a wide range of therapeutic payloads with high precision [10,11,12]. By exploiting their receptor selectivity, favorable pharmacokinetics, and tunable physicochemical properties, peptides can be conjugated to nanoparticles, cytotoxic small-molecule drugs, or radionuclides to enhance delivery, improve tumor retention, and minimize off-target toxicity. These peptide-guided systems bridge the gap between molecular recognition and therapeutic potency, enabling the selective transport of chemotherapeutics across physiological barriers, the activation of controlled drug release, and the targeted deposition of ionizing radiation within malignant tissues [13,14]. Together, peptide-functionalized nanocarriers, peptide–drug conjugates, and peptide-based radionuclide constructs constitute a rapidly expanding class of precision therapeutics designed to overcome limitations of conventional chemotherapy and biologics, including poor tumor penetration, systemic toxicity, and lack of specificity. The following subsections summarize the mechanistic principles, design strategies, and translational progress of these peptide conjugate modalities in oncology.

### 2.1. Nanocarrier Peptide Systems

Tumor-homing peptides (THPs) are short amino acid sequences that selectively recognize tumor cells or components of the tumor microenvironment, enabling the targeted delivery of therapeutics or imaging agents while minimizing off-target effects [8]. Their conjugation to drug delivery platforms—including synthetic nanoparticles (NPs) and biologically derived extracellular vesicles (EVs), particularly exosomes—enhances stability, binding avidity, and therapeutic precision [8,9]. NP-based systems have markedly improved therapeutic precision in oncology by enabling surface functionalization with targeting ligands, thereby increasing drug accumulation at tumor sites while reducing systemic toxicity. Among available nanoplatforms, iron oxide magnetic NPs are particularly attractive due to their biocompatibility, straightforward synthesis, and the versatility of metallic or polymeric coatings that facilitate efficient functionalization [9]. Peptides serve as ideal NP ligands because of their high specificity, tunable affinity, and chemical adaptability; however, conjugation strategies such as metal-ion chelation, Au–thiol bonding, or maleimide–thiol coupling must be carefully optimized, as multiple reactive groups can lead to uncontrolled attachment and compromised bioactivity. The incorporation of non-natural functional groups can improve conjugation selectivity and maintain peptide orientation and stability, thereby preserving the therapeutic performance of peptide-modified NP systems [15]. Beyond synthetic nanocarriers, THPs have also been integrated into natural delivery systems such as EVs and exosomes, which offer intrinsic advantages including high biocompatibility, low immunogenicity, and the innate ability to cross biological barriers [16]. Exosomes are nanosized vesicles of endocytic origin released upon fusion of multivesicular bodies with the plasma membrane and play a central role in intercellular communication by transferring endogenous cargo—DNA, RNA, proteins, and lipids. Their lipid bilayer and bioactive payload can be further engineered to improve targeting specificity and therapeutic efficiency, making them an increasingly attractive complement to synthetic nanocarriers [8,16].

Many THPs are used across NP and exosome platforms; thus, the following section integrates the main THPs used in oncology. This section highlights how each peptide has been exploited in NP-based and exosome-based systems to enhance tumor selectivity, cellular internalization, and overall antitumor efficacy.

**Figure 1:**
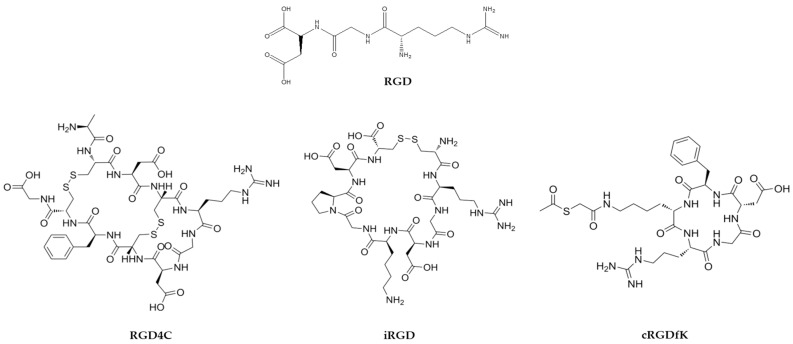
Chemical structure of the RGD peptide and its derivatives RGD4C, iRGD, and cRGDfK.

Among THPs investigated for NP functionalization, RGD peptides remain one of the most extensively studied examples. Composed of the arginine–glycine–aspartic acid motif, they bind with high affinity to αvβ3 and αvβ5 integrins, which are overexpressed in tumor vasculature and cancer cells [17]. In addition to mediating targeting, RGD peptides can modulate intracellular signaling pathways, in some cases contributing to resistance of cancer cells to antiproliferative drugs [17]. Their dual role in targeting and signaling renders RGD peptides valuable tools for enhancing the specificity and efficacy of NP-based drug delivery systems [17]. A wide range of NP formulations have been functionalized with RGD motifs—including polydopamine NPs evaluated in thyroid cancer, liposomes in BC and glioma, gold NPs in BC, lung, and melanoma models, and polymeric nanocarriers in glioblastoma xenografts—consistently demonstrating enhanced tumor accumulation and improved therapeutic outcomes in preclinical studies [18,19,20,21,22]. Figure 1 depicts the chemical structure of the RGD peptide and their derivatives.

Recent advances further highlight the versatility of RGD-based peptides to improve stability, specificity, and tumor penetration. By exploiting integrin overexpression in malignant tissues, RGD motifs have been incorporated into nanodrug formulations and conjugates to enhance tumor penetration and therapeutic precision. Several studies demonstrated that RGD-modified NPs improve selective tumor targeting in preclinical models (reviewed in [23]), and RGD-based self-assembling nanodrugs achieve superior tumor penetration and efficacy (reviewed in [19]). Translational insights underscore the clinical potential of integrin-targeted RGD applications while also addressing challenges such as off-target uptake and heterogeneity of integrin expression [24].

Several engineered derivatives of the RGD motif have been engineered to improve stability, specificity, and tumor penetration. Cyclic derivatives, including RGD4C, cRGDfK, and iRGD, display improved conformational rigidity and enhanced binding specificity relative to linear peptides. Among these, RGD4C and iRGD (Figure 1) are particularly well characterized. RGD4C (ACDCRGDCFCG), originally isolated through phage-display technology, selectively enters tumor cells with high αvβ3 expression, with no detectable uptake in normal cells [17]. Fluorescence studies show sustained intracellular accumulation following internalization. In addition to free-peptide applications, RGD4C has been widely incorporated into nanocarriers to enhance stability and targeting precision [17]. For example, RGD4C-decorated polymeric NPs (PEO-b-P(CL-Hyd-DOX)) showed enhanced tumor accumulation and improved therapeutic efficacy in xenograft models compared to non-decorated NPs or free drugs [25]. Similarly, RGD4C-modified apoferritin nanocages loaded with doxorubicin (DOX) exhibited selective homing to αvβ3-expressing tumors and high drug encapsulation efficiency [26].

The cyclic pentapeptide cRGDfK (Figure 1) has been investigated as a carrier for radioactive payload in BC, which is particularly relevant in patients resistant to HER2-directed therapy [27]. Preclinical studies in mice showed effective tumor reduction using cRGDfK dimers. However, dosimetric analysis indicated that the kidneys exhibited the highest absorbed radiation dose, consistent with renal clearance of peptide-based agents [27].

The iRGD peptide is a cyclic nonapeptide (CRGDCKGDC) derived from RGD motif and specifically engineered to enhance tumor penetration [8]. It first binds αvβ3/αvβ5 integrins and subsequently exposes a C-end rule (CendR) motif that engages neuropilin-1 (NRP-1) and initiates active transport, vascular extravasation, and deep intratumoral penetration [8,28]. Importantly, several RGD peptides have been evaluated in clinical trials focused on various types of cancer (extensively reviewed in [24]). Across different nanoplatforms, these mechanisms consistently translate into more efficient drug deposition within tumors. For example, in BC and pancreatic cancer models, iRGD-functionalized NPs increased intratumoral drug delivery, reduced metastatic burden, and prolonged survival [8,16,28]. Building on this, silica-based nanocarriers decorated with iRGD further exploited NRP-1-mediated transport to enhance the penetration of gemcitabine or nab-paclitaxel, achieving synergistic antitumor effects in pancreatic tumors [25]. Also, lipid–polymer hybrid NPs showed comparable advantages in BC, where iRGD modification improved tissue penetration and therapeutic response [26]. The benefits of iRGD derivatives extend to biologically derived systems. iRGD-modified exosomes improved DOX loading efficiency, tumor targeting, and intratumoral accumulation in BC models [22]. Similarly, exosomes engineered to display iRGD through LAMP2B fusion in human embryonic kidney (HEK-293T) cells effectively delivered DOX to anaplastic thyroid carcinoma, leading to robust tumor-suppressive activity in vitro and in vivo [29]. Altogether, these findings underscore the versatility of iRGD as a potent enhancer of tumor targeting and penetration across both synthetic nanocarriers and exosome-based delivery systems. These studies position RGD-derived peptides within the broader class of delivery and targeting peptides, with overlaps into peptide–drug conjugates (PDCs) when conjugated to cytotoxic or immunomodulatory agents, thereby exemplifying their dual role as both carriers and active therapeutic enhancers.

Beyond RGD variants, additional THPs have broadened the applicability of peptide-guided nanocarriers and vesicle-based systems. One notable example is PO-6, an antagonistic peptide that targets CD123 (IL-3Rα), a receptor overexpressed in multiple hematological malignancies [30]. Developed through a fragment-based cell screening approach, PO-6 was incorporated into micelles using an amphipathic polymer (^m^PO-6) to enhance its solubility and stability. This peptide-guided nanocarrier demonstrated high selectivity for CD123^+^ acute myeloid leukemia (AML) cells. In these cells, the peptide effectively disrupted CD123/IL-3 signaling, leading to inhibition and downstream STAT-5, PI3K/AKT, and NF-κB pathways [30]. In vivo, mPO-6 reduced leukemic infiltration and significantly prolonged survival in AML mouse models, illustrating the therapeutic potential of THP-functionalized NP systems in hematological cancers.

Beyond hematological targets such as CD123, several tumor-homing peptides with broader applicability have been integrated into both nanoparticle- and vesicle-based delivery systems. Among these, LyP-1 stands out as one of the most extensively explored ligands. LyP-1 is a cyclic nine-amino-acid peptide (CGNKRTRGC) that exhibits potent tumor and lymphatic homing properties. Originally developed for BC, it preferentially accumulates in the nuclei of primary and metastatic tumor cells and can induce apoptosis. Mechanistically, it binds with p32, a mitochondrial protein aberrantly expressed at the surface of tumor cells. Also, it targets markers of lymphatic angiogenesis such as VEGF-C and invasion-associated proteins as MMP2. These properties have motivated the incorporation of Lyp-1 into diverse NP formulations. Examples include LyP-1-functionalized liposomal or lipidic nanosystems carrying DOX, which enhanced tumor accumulation and reduced tumor growth in BC models [31,32]. Similarly, LyP-1-functionalized chitosan NPs carrying endostatin achieved stronger p32-mediated targeting and enhanced antiangiogenic activity [33]. Alternative NP platforms such as multi-walled carbon nanotubes conjugated with LyP-1 for siRNA delivery in pancreatic cancer have shown cellular uptake and robust tumor suppression [34]. LyP-1 has also been successfully integrated into biologically derived carriers. LyP-1-modified exosomes display enhanced tropism for lymphatic and hypoxic tumor niches and efficiently deliver poorly soluble chemotherapeutics or pro-apoptotic agents. Ferritin nanocages engineered with the truncated tLyP-1 engineered ferritin nanocages to encapsulate paclitaxel improved penetration in 3D tumor spheroids and increased antitumor efficacy in vivo [35]. The truncated tLyP-1 functionalization also enables binding to C1QBP (p32) and interaction with NRP-1/2, conferring strong tumor-penetrating properties and enhanced apoptotic activity [35].

Building on the versatility demonstrated by peptides described, across synthetic and biological carriers, additional THPs have expanded the applicability of peptide-guided nanocarriers, EVs, and exosomes. Among these, GE11 and T7 represent two well-established ligands engineered to target key cancer-associated receptors with high specificity. The GE11 peptide (YHWYGYTPQNVI), a synthetic peptide that binds epidermal growth factor receptor (EGFR) without activating downstream proliferation signals, has been displayed on EVs derived from human umbilical vein endothelial cells (HUVECs). GE11-engineered EVs selectively targeted EGFR overexpression in lung cancer models, where they inhibited angiogenesis, reduced carcinoma progression, and promoted T lymphocyte recruitment to the spleen [36]. These findings highlight GE11 as a non-mitogenic alternative to EGF for receptor-directed delivery. Similarly, the T7 peptide (HAIYPRH) is a high affinity for the transferrin receptor (TfR), which is overexpressed in several tumors, including glioblastoma (GBM), and plays a central role in iron uptake across the blood–brain barrier (BBB) [37]. To leverage this pathway, T7 was fused with Lamp2b to engineer T7-modified exosomes capable of selectively binding to GBM cells. These T7 exosomes effectively deliver cholesterol-modified siYY1, a small interfering RNA (siRNA) targeting transcription factor YY1, leading to its knockdown and a significant inhibition of GBM growth in preclinical models. This strategy underscores the potential of T7-functionalized exosomes to overcome the BBB and achieve precise delivery to brain tumors [37]. In parallel with peptide engineering, it has become increasingly clear that exosomes themselves actively modulate the tumor microenvironment. For example, in cancers undergoing Snail-induced epithelial-to-mesenchymal transition (EMT), tumor cells release microRNA-21 (miR-21) via exosomes after chemotherapy. This exosomal miR-21 suppresses inflammasome activation in tumor-associated macrophages (TAMs), thereby promoting immune evasion and contributing to therapeutic resistance [38]. The integration of THP with exosome-based delivery systems represents the most promising strategies in targeted cancer therapy, combining the natural biocompatibility and low immunogenicity of EVs with the molecular precision of peptide targeting. Table 1 shows examples of tumor-homing peptides used for guided delivery of exosomes to tumor cells.

Overall, current evidence demonstrates that THPs can substantially improve the precision and efficacy of NP- and exosome-based delivery systems, particularly when targeting integrins (iRGD), p32-positive tumor niches (LyP-1/tLyP-1), and lineage-specific receptors such as CD123 (mPO-6) or EGFR (GE11). Among these ligands, iRGD and LyP-1 variants show the most consistent performance, repeatedly enhancing intratumoral penetration, drug accumulation, and antitumor activity across multiple tumor models [8,16,26,28,31,32,33,34,35]. Similarly, receptor-specific ligands like GE11 or T7 have demonstrated compelling results in preclinical EV platforms, but their effectiveness is strongly dependent on the expression profile of EGFR or TfR, which may vary widely across patient subgroups. Taken together, these findings indicate that while several THPs reliably enhance targeting and penetration, the success of peptide-guided delivery remains tightly linked to receptor accessibility, tumor heterogeneity, and the biological properties of the chosen carrier. Although translational challenges such as immunogenicity, pharmacokinetics, and large-scale manufacturing will be addressed in a later section, it is already evident that THPs with dual-function mechanisms—such as binding plus active transport (iRGD) or simultaneous targeting of hypoxic and lymphatic niches (LyP-1/tLyP-1)—offer the strongest foundation for clinical translation. Importantly, many peptides have also been adapted beyond carrier surface functionalization and are now being incorporated into peptide–drug conjugates, a strategy that further exploits their specificity while enabling direct payload coupling.

### 2.2. Peptide Guide Radionuclides

As peptide engineering continues to expand the scope of precision oncology, their integration into radionuclide-based platforms represents a logical evolution. In this context, peptides function as molecular homing devices that deliver diagnostic or therapeutic isotopes with high spatial selectivity, enabling more accurate tumor imaging and effective targeted radiotherapy. Peptide receptor radionuclide therapy (PRRT) combines tumor-specific peptides with radionuclides to achieve preferential accumulation of radioactivity within malignant tissues while sparing surrounding healthy organs. By binding receptors that are overexpressed on cancer cells, peptide–radionuclide conjugates internalize into the target tissue, where emitted radiation induces DNA double-strand breaks and irreversible apoptotic cell death [39,40]. This strategy provides a potent therapeutic option for patients with advanced, metastatic, or surgically unresectable tumors and improves the therapeutic efficacy relative to non-targeted radiotherapy [41].

Radionuclides used in PRRT are selected to balance effective cytotoxicity with limited tissue penetration, thereby optimizing tumor control while minimizing toxicity. The most widely implemented isotopes include iodine-131 (^131^I), yttrium-90 (^90^Y), lutetium-177 (^177^Lu), and, to a lesser extent, rhenium-188 (^188^Re) [42]. Among these, ^90^Y emits high-energy β-particles capable of penetrating deeper tumor regions. Nevertheless, it is suitable for larger lesions and it is also associated with an increase in renal exposure. Conversely, ^177^Lu emits β-particles with an intermediate energy and shorter range, offering a more favorable safety profile and reduced nephrotoxicity, which has led to its widespread clinical adoption [43]. Several peptide–radionuclide constructs have been evaluated across preclinical and clinical settings (Table 2), further illustrating the versatility and translational potential of peptide-guided radionuclides.

Among the various peptide–receptor systems explored in PRRT, the somatostatin receptor family (SSTRs) represents the most established and clinically validated target. Somatostatin is an endogenous peptide that regulates neuroendocrine and neuronal functions through five G-protein-coupled receptors (SSTR1-5). Among these, SSTR2 is the most overexpressed in neuroendocrine neoplasms (NENs). High SSTR2 density is characteristic of many NENs subtypes, including growth hormone-secreting pituitary adenomas and thyroid-stimulating hormone-secreting tumors, enabling the efficient and selective accumulation of radiolabeled somatostatin analogues [42]. First-generation synthetic somatostatin analogues (SSAs) such as octreotide and lanreotide were developed to mimic the binding affinity of native somatostatin while providing greater metabolic stability and longer half-life. These analogues constitute first-line therapies for functional NENs, where they effectively reduce hormone hypersecretion and slow tumor progression with a favorable safety profile [42,43]. Their high affinity for SSTR2 also renders them ideal scaffolds for PRRT development. Nevertheless, PRRT has also been expanded to alternative tumor-associated receptors. Below we describe some notable examples.

One example is ^117^Lu-FAP-2286, a peptide targeting the fibroblast activation protein (FAP), which is overexpressed in stromal compartments of mesenchymal and epithelial tumors, including sarcoma and mesothelioma. Early clinical studies have reported selective tumor accumulation and preliminary antitumor activity in pancreatic, ovarian, and colorectal cancers [44]. Similarly, ^177^Lu-PSMA conjugates exploit the prostate-specific membrane antigen (PSMA), a type II transmembrane protein that enhances tumor cell survival and proliferation. This conjugate has shown promise as theranostic agents in prostate cancer owing to the favorable half-life (6–7 days) and emission profile of ^177^Lu [45]. Additionally, ^90^Y-labeled peptide constructs have been explored for the treatment of colorectal liver metastasis cancer. These constructs target receptors overexpressed on metastatic lesions and are delivered via chelators such as DOTA (1,4,7,10-tetraazacyclododecane-1,4,7,10-tetraacetic acid). Sequential lobar radioembolization with ^90^Y has yielded modest survival improvements and demonstrated low toxicity in dedicated clinical devices [46].

Octreotide, an eight-amino-acid cyclic peptide (FCFWKTCT), served as the backbone for early radiolabeled PRRT agents [42]. The first clinically tested construct, [^90^Y]-DOTATOC (OctreoTher^TM^, Novartis), demonstrated the ability to stabilize diseases in patients with NENs while maintaining acceptable tolerability with mild side effects fatigue or nausea. However, renal and bone marrow toxicity were identified as dose-limiting factors. Comparative studies revealed that [^90^Y]-DOTATATE exhibits higher in vitro affinity for SSTR2, whereas [^111^In]-DOTATOC provides superior imaging contrast in humans. Despite these functional differences, their therapeutic efficacy is broadly similar, and PRRT has been associated with substantial survival benefits with median overall survival reaching ~50 months in comparison with ~18 months in untreated patients. Long-term follow-up confirms that SSTR-targeted PRRT with ^90^Y-labeling is generally well tolerated and capable of inducing durable disease control [47,48,49,50]. Nevertheless, robust phase III clinical trial data are emerging. The pivotal NETTER-1 Phase 3 trial evaluated ^177^Lu-DOTATATE in patients with advanced midgut neuroendocrine tumors and provided the strongest clinical evidence supporting PRRT. In this randomized study, treatment with ^177^Lu-DOTATATE significantly improved progression-free survival compared to high-dose octreotide, with median PFS not reached in the PRRT arm versus 8.4 months in the control group. Objective response rates were also higher, and the therapy demonstrated a favorable safety profile, with manageable hematologic and renal toxicities. Importantly, overall survival analyses indicated a substantial survival benefit, establishing ^177^Lu-DOTATATE as a standard of care for patients with progressive, somatostatin receptor-positive midgut neuroendocrine tumors [51]. More recently, ^177^Lu-HA-DOTATATE, a next-generation somatostatin analogue radiolabeled with lutetium-177, has been evaluated in clinical trials. This construct was designed to improve tumor retention and therapeutic index relative to conventional ^177^Lu-DOTATATE while maintaining a favorable safety profile. Early-phase studies demonstrated selective uptake in SSTR2-positive lesions, durable disease control, and manageable toxicity, reinforcing the role of lutetium-based PRRT as the current standard of care in neuroendocrine tumors [41].

Although PRRT has demonstrated clear clinical value across several tumor types, important differences emerge when comparing the therapeutic profiles of individual peptide–radionuclide platforms. SSTR-targeted constructs remain the most clinically validated, supported by decades of safety data and by the favorable therapeutic index of ^177^Lu-labeled analogues. They consistently outperform high-energy ^90^Y formulations in terms of toxicity profile and durability of response, while ^90^Y-based agents offer deeper tissue penetration and can be advantageous in large, bulky lesions. Also, they are associated with increased renal and marrow exposure, limiting their therapeutic window. In contrast, ^177^Lu-DOTATATE and related SSTR2-directed agents achieve a more balanced combination of tumor retention, internalization efficiency, and controlled β-particle range—factors that explain their superior long-term disease control rates and widespread clinical adoption. Emerging systems such as FAP-2286 or PSMA-targeted ^177^Lu conjugates broaden the scope of PRRT beyond neuroendocrine malignancies. Yet their clinical impact remains preliminary, with early signs of antitumor activity but without the robust survival evidence available for SSTR-based PRRT. Collectively, these comparisons highlight that therapeutic performance in PRRT is determined not only by isotope choice but also by receptor density, internalization kinetics, off-target expression, and peptide scaffold stability. These parameters dictate which constructs translate successfully from preclinical promise to durable clinical benefit. The advances of PRRT in patients with advanced neuroendocrine tumors and possible strategies to combine this therapy with locoregional and systemic anticancer treatments are reviewed in [52,53].

### 2.3. Peptide–Drug Conjugates (PDCs)

**Figure 2:**
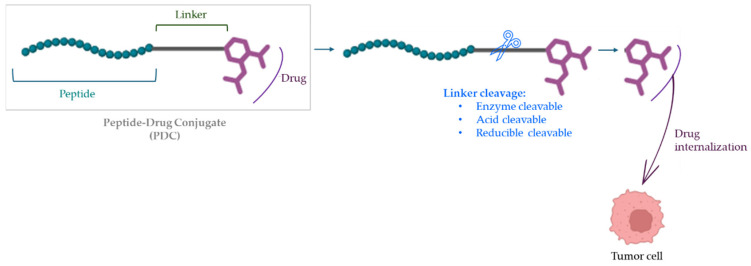
Schematic representation of a peptide–drug conjugate (PDC), showing its modular architecture (peptide, linker, and drug) and the mechanism of linker cleavage—enzyme-, acid-, or reduction-sensitive—leading to drug release and internalization in tumor cells (Created in BioRender.com. https://app.biorender.com/illustrations/61533a6e5634fc00a6af13ce (accessed on 16 December 2025)).

Peptide–drug conjugates (PDCs) represent an emerging class of targeted therapeutics that integrate the specificity of peptides with the cytotoxic potential of small-molecule drugs, enabling precise delivery to tumor tissues while minimizing systemic toxicity. Structurally, a PDC consists of three key components: (i) a targeting peptide with high affinity for tumor-associated receptors; (ii) a linker which dictates the stability and release kinetics of the drug, and (iii) a cytotoxic payload, typically a potent chemotherapeutic agent (Figure 2) [4,8,54]. This modular architecture enhances pharmacokinetic control, promotes tumor penetration, and enables the use of highly toxic drugs that are otherwise unsuitable as free agents while minimizing off-target effects. Compared with larger delivery systems such as NPs or exosomes, PDCs benefit from small molecules. This improves tissue diffusion and allows crossing of physiological barriers, including in some cases the blood–brain barrier (BBB) [4,54]. Their synthetic uniformity also facilitates reproducibility and manufacturing scalability, supporting their translational potential. This conceptual framework has been extensively reviewed, highlighting PDCs as a next-generation alternative to antibody–drug conjugates, with unique advantages in tumor penetration and modularity [55].

A central determinant of PDC activity is linker chemistry. Linkers mut remain sufficiently stable in circulation to prevent premature drug release yet must be cleavable under tumor-specific stimuli. Common linker categories include enzyme-sensitive, acid-labile, redox-responsive (e.g., disulfide bonds), and non-cleavable linkages [54]. Recent analyses of clinically advanced PDCs show extensive use of amide, triazole, carbamate, sulfonamide, ester, and disulfide linkers. Each of these linkers provides distinct stability and release profiles (e.g., disulfides for intracellular glutathione-mediated cleavage; carbamates for protease-triggered release) [56]. The choice of linker is critical: it must remain stable during circulation to avoid premature drug release while allowing efficient liberation at the target site [54]. This modular architecture allows rational optimization of each component to enhance specificity, potency, and safety. This chemical versatility has supported the rapid expansion of PDC architecture across oncology and beyond. Recent analyses confirm the rapid diversification of PDC architectures and their clinical progress, underscoring the importance of rational linker design and payload selection (reviewed in [12]). Below we describe some examples of PDCs.

NGR peptides (Asn-Gly-Arg) represent another important class of vascular-homing ligands. They bind aminopeptidase N (CD13), a zinc-dependent metalloprotease involved in proliferation, angiogenesis, and tumor invasion [57,58]. NGR peptides have been explored for targeting delivery of different compounds to tumors, including a cytotoxic agent, such as Daunomycin (Dau) via oxime linkage. In preclinical models using HT-1080 fibrosarcoma and HT-29 colorectal cancer cells, the cyclic NGR peptide with Dau showed efficient cellular uptake and strong cytostatic and cytotoxic activity [58].

For instance, cell surface keratin 1 (K1), overexpressed in several epithelial cancers (e.g., neuroblastoma, nasopharyngeal, and hepatocellular carcinomas), contributes to tumorigenesis and apoptosis dysregulation and has emerged as an attractive target. The BC-targeting peptide 18-4 (WxEAAYQrFL) shows selective internalization in tumor cells without detectable uptake in normal cells, enable its use for PDC design [59]. Conjugates based on 18-4 demonstrate efficient endocytosis and improved tumor selectivity, highlighting the use of receptor-mediated peptide internalization for precise drug delivery [59].

PDCs can also be directed toward elements of the tumor microenvironment. The peptide CREKA (Cys-Arg-Glu-Lys-Ala) binds to fibrin–fibronectin complexes enriched in tumor vasculature and stroma [60]. Conjugation of CREKA to PEGylated liposomes loaded with DOX (CREKA-lipo-DOX) resulted in selective homing to tumor tissue. This conjugation induces a significant reduction in metastasis and increased therapeutic efficacy in models of breast and lung cancer [60]. These findings demonstrate that targeting stromal components can complement direct tumor cell targeting, particularly in desmoplastic or heterogeneous tumors.

Similar strategies exploit overexpressed hormonal receptors, such as the luteinizing hormone-releasing hormone (LHRH) receptor (also known as gonadotropin-releasing hormone (GnRH)), to further enhance drug accumulation and therapeutic outcomes. LHRH, a decapeptide (GHTSTGLAPG) that regulates gonadal hormone production, is overexpressed in 74% of BC cases, which renders it a clinically validated targeting motif [61]. For example, an LHRH-targeted co-delivery system for DOX and dasatinib (Das) achieved synergistic cytotoxicity in resistant triple-negative BC, underscoring the utility of peptide-guided strategies in addressing drug resistance [61]. Notably, the clinically advanced PDC AEZS-108 (zoptarelin-DOX), a conjugate of LHRH and DOX, progressed to phase III trials for endometrial cancer (NCT01767155). Despite the promising results in phase I/II, in phase III, this PDC failed to improve patient survival [62,63].

Another example involves APRPG (Ala-Pro-Arg-Pro-Gly), a synthetic peptide selective for vascular endothelial growth factor receptor-1 (VEGFR-1) expressed in angiogenic blood vessels [64]. APRPG-modified liposomal co-loaded with paclitaxel (PTX) and norcantharidin significantly inhibited angiogenesis and induced apoptosis in hepatocellular carcinoma models, achieving a tumor growth inhibition rate of 78.7% [8,64].

Recent database analyses show that PDCs encompass hundreds of unique combinations of peptides, linkers, and drugs, reflecting a rapidly diversifying field. Many PDCs use classical chemotherapeutics as payloads, including DOX, PTX, daunorubicin, methotrexate, camptothecin, vincristine, and platinum derivatives [56]. In fact, there is an expanding interest in stimuli-responsive linkers, CPP-based PDCs, and conjugates engineered to optimize tissue tropism, receptor internalization rates, and intracellular trafficking, strengthening the mechanistic foundation for next-generation PDC design [65].

These examples illustrate only a fraction of the rapidly expanding landscape of PDCs and highlight how therapeutic performance varies substantially depending on the targeting motif, payload potency, and linker stability. K1-targeting constructs such as peptide 18-4 offer strong internalization efficiency and excellent tumor selectivity, but their efficacy remains largely preclinical. Stroma-directed systems such as CREKA-based conjugates achieve robust antimetastatic effects, particularly in desmoplastic tumors. Nevertheless, their therapeutic impact depends heavily on stromal abundance and fibrin–fibronectin accessibility. Hormone receptor-targeted PDCs, exemplified by LHRH-based formulations including AEZS-108, have shown high tumor uptake and promising early responses, but the failure of AEZS-108 in phase III trials underscores the difficulty of translating strong uptake into survival benefit. In contrast, vascular-targeting peptides such as APRPG demonstrate potent antiangiogenic activity and high tumor growth inhibition, but their efficacy is tightly linked to angiogenic status and may vary across tumor types. Altogether, these comparisons emphasize that no single PDC architecture is universally superior. Efficacy is dictated by the interplay between receptor density, internalization kinetics, linker chemistry, and payload potency, and the most successful candidates are those in which all three components are optimally aligned for a given tumor context.

## 3. Peptides with Intrinsic Antitumor Activity

Peptides with intrinsic antitumor activity represent a mechanistically diverse class of therapeutics whose effects arise directly from their biochemical and biophysical properties. Unlike the peptide-guided carriers discussed previously, these molecules act as active agents, not delivery vectors. They exert cytotoxic or regulatory functions through multiple pathways including receptor agonism or antagonism, inhibition of oncogenic protein–protein interaction (PPIs), membrane disruption, mitochondrial destabilization, and direct modulation of intracellular signaling [66].

### 3.1. Peptide Antagonists/Agonists of Receptor Tyrosine Kinases and Hormone Receptors

In oncology, peptide antagonists have been developed for a variety of applications aiming to disrupt signaling pathways that sustain tumor growth or immune evasion (see Section 4) [66]. Among these targets, receptor tyrosine kinases (RTKs), type I transmembrane protein receptors that play essential roles in regulating intercellular communication, proliferation, cell survival and metabolism, migration, and cell cycle control, stand out [67]. Ligand binding induces RTK dimerization, followed by autophosphorylation of cytoplasmic tyrosine residues. This phosphorylation initiates downstream signaling cascades by creating docking sites for adaptor proteins containing phosphotyrosine-binding domains [68]. Dysregulation of RTK signaling is a hallmark of multiple cancers, underscoring their relevance as therapeutic targets [67]. The subsequent examples illustrate peptide antagonists directed against RTKs.

AXL belongs to the TAM family of RTKs and is activated by its high-affinity ligand GAS6, promoting pathways that sustain cancer cell survival, proliferation, migration, and invasion. This interaction promotes signaling pathways that drive cancer cell survival, proliferation, migration, and invasion [68]. Structurally, AXL comprises two immunoglobulin-like repeats and two fibronectin type III repeats, and intracellular kinase domain responsible for autophosphorylation and downstream signaling. TAM receptors are expressed in diverse tissues and cell types, including monocytes, platelets, endothelial cells, the cerebellum, and the liver, where they regulate cell survival, differentiation, and platelet aggregation [68]. *AXL* gene expression is controlled by multiple transcription factors, and its dysregulation varies across cancer types. For instance, MET activation induces AXL mRNA expression in urothelial carcinoma, linking the receptor to tumor progression and therapeutic resistance [68]. Given its oncogenic role, the GAS6-AXL axis has been directly targeted by peptide-based antagonists. Recent studies and patents describe engineered peptides derived from the extracellular domain of AXL, designed to bind GAS6 with higher affinity than native AXL, thereby competitively blocking ligand binding and downstream signaling. These peptide antagonists—sometimes formulated as peptide–polymer conjugates or Fc-fusion constructs—have inhibited AXL signaling and reduce tumor growth and metastasis in preclinical models, thus providing proof-of-concept for peptide-guided extracellular blockade of AXL in oncology [69,70].

Discoidin domain receptors DDR1 and DDR2 are members of the RTK family uniquely activated by collagen, a major extracellular matrix component. DDR1 is broadly expressed in multiple tissues, and DDR2 is restricted to mesenchymal-type cells [71]. Dysregulation of DDR1—through overexpression or mutation—has been implicated in the progression of several lungs, breast, brain, liver, pancreas, and prostate malignancies. Also, it has been implicated in non-malignant diseases associated with chronic inflammation [71]. Through their collagen-binding activity, DDRs modulate tumor–stroma communication, supporting tumor growth and progression. At the molecular level, the domain of DDRs contains an ATP-binding pocket flanked by two lobes. In the inactive state, the activation loop blocks this site prevents phosphorylation [71]. Several small-molecule inhibitors targeting DDR1/DDR2 have been developed, but they exhibit off-target activity, which remains a limitation due to their similarity of their ATP-binding domains with other kinases [71]. Peptide-based inhibitors include DDR2-derived functional domain peptides delivered via Au-NP-DNA aptamer conjugates, which abrogate collagen-induced DDR2 activation and inhibit lung cancer cell proliferation and invasion. Additionally, collagen-mimetic peptide decoys reproducing the GVMGFO motif can also block DDR1–collagen interactions, though current evidence is largely in vitro [72]. Genetic silencing (RNAi) or small-molecule inhibition of DDR1 has already been shown to impair tumor progression, validating DDR1 as a promising therapeutic target [73,74]. In parallel, other RTKs, such as DYRK1A, are under investigation. Inhibition of DYRK1A enhances β-cell differentiation and survival, and preclinical studies suggest potential anticancer activity, although this remains at an exploratory stage [75].

Other clinically relevant RTKs have also been investigated as peptide targets. Among them, the epidermal growth factor receptor (EGFR) represents a prototypical RTK widely implicated in epithelial cancers (e.g., lung, breast, colorectal, and glioblastoma). Structurally, EGFR comprises an extracellular ligand-binding domain, a single transmembrane helix, and an intracellular tyrosine kinase domain. Upon binding of its ligands (e.g., EGF, TGF-α), EGFR induces receptor dimerization and autophosphorylation, triggering downstream signaling through the RAS–RAF–MEK–ERK and PI3K–AKT pathways that promote proliferation and survival. Aberrant EGFR activity, through overexpression or mutation, is a major oncogenic driver in several solid tumors. Among peptide-based strategies, Disruptin has emerged as a notable example. This biotinylated nonadecapeptide composed of an 8–amino acid segment from the EGFR αC-helix–β4 sheet loop (residues S767–C774) fused to an 11–amino acid TAT sequence [76]. The design enables cellular uptake and interferes with EGFR dimerization. This attenuating downstream signaling and reducing clonogenic survival of EGFR-dependent tumor cells in vitro and in xenograft models [77].

Following EGFR, other receptor tyrosine kinases have been investigated as peptide targets, particularly those involved in angiogenesis and growth factor signaling. Vascular endothelial growth factor receptors (VEGFR1–3) are central mediators of tumor vascularization. They are activated by VEGF ligands, leading to endothelial proliferation, migration, and neovascularization, processes critical for tumor growth and metastasis. Pathological upregulation of VEGF–VEGFR signaling is a hallmark of many solid tumors, making VEGFR blockade a major therapeutic strategy. In this context, peptide antagonists such as F56 (WHSDMEWWYLLG) have been shown to block VEGF–VEGFR interactions. This blocking inhibits endothelial migration and tube formation, suppressing tumor vascularization in vivo [78]. The rationally designed peptide VGB4 mimics discontinuous binding sites of VEGF-A and VEGF-B. This peptide binds to VEGFR1 and VEGFR2 with high affinity, effectively blocking receptor signaling and inducing tumor regression in murine models [79].

Beyond angiogenesis, the insulin-like growth factor 1 receptor (IGF-1R) represents another clinically relevant RTK. IGF-1R is strongly implicated in cancer development, therapy resistance, and poor prognosis, particularly in breast and prostate cancers. Its activation promotes proliferation and survival via the PI3K/AKT and MAPK cascades. While numerous inhibitors have been developed against IGF-1R—mainly monoclonal antibodies and small molecules—peptide-based antagonists remain largely unexplored, with no validated candidates currently available [80,81]. This gap highlights an opportunity for future peptide-engineering approaches to expand the therapeutic repertoire against IGF-1R-driven tumors.

Beyond growth factor receptors, hormone-dependent cancer represents other major context in which peptide-based strategies have been explored. Hormone-dependent cancers rely on specific hormones, and therapeutic approaches focus on inhibiting either hormone production or hormone–receptor interactions, thereby suppressing tumor proliferation. Hormone therapy remains the cornerstone of treatment for breast and prostate cancers, administered as neoadjuvant or adjuvant therapy to reduce tumor burden, improve surgical outcomes, and lower recurrence risk [8]. Among peptide-based hormone receptor modulators, gonadotropin-releasing hormone (GnRH), also known as luteinizing hormone-releasing hormone (LHRH), is one of the most extensively studied. GnRH is an endogenous decapeptide produced in the hypothalamus that regulates reproductive function by stimulating pituitary gonadotropes to release luteinizing hormone (LH) and follicle-stimulating hormone (FSH). It has also been described as a PDC, showing, once again, the versatility of peptides. Two isoforms exist in mammals, with GnRH1 (QHWSYGLRPG) being the predominant hypothalamic isoform. Importantly, GnRH receptors are aberrantly expressed in peripheral tissues, including prostate, kidneys, and bone marrow, where they have been implicated in tumor biology [82]. Therapeutically, synthetic GnRH analogues have been developed as agonists and antagonists, providing the foundation for androgen deprivation therapy (ADT) in prostate cancer and endocrine modulation in BC (Table 3).

GnRH agonists (e.g., leuprolide, goserelin, triptorelin, and buserelin) activate pituitary GnRH receptors, leading to a transient rise in LH, FSH, and testosterone, a phenomenon known as tumor flare effect [82]. With continuous administration, gonadotropin release declines and testosterone reaches castrate levels. To counteract the flare phenomenon in advanced prostate cancer, agonists are frequently co-administered with antiandrogens such as bicalutamide [83]. In contrast, GnRH antagonists (e.g., degarelix, cetrorelix, and the oral non-peptide relugolix) competitively block pituitary GnRH receptors, producing an immediate decline in testosterone without the flare response [84]. This results in reduced androgen receptor (AR) activity, disruption of growth factor receptor crosstalk, and modulate of downstream signaling pathways such as Gαi–cAMP, ultimately inducing apoptosis and suppression tumor proliferation [85]. Clinically, agonists and antagonists form the backbone of ADT for advanced and metastatic prostate cancer. Agonists remain widely used for their established efficacy, while antagonists offer faster testosterone suppression and lower flare risk. Notably, the Phase 3 HERO trial (NCT03085095) showed that relugolix enables faster testosterone recovery after discontinuation. Additionally, it is associated with fewer cardiovascular events compared to leuprolide, highlighting its potential as a safer long-term alternative [86]. At the structural level, GnRH analogues incorporate modifications such as D-amino acid substitution at position 6 and alterations in the C-terminal region (i.e., positions 9–10) to enhance receptor affinity, half-life and reduce metabolic degradation. Collectively, preclinical and clinical evidence confirms that GnRH-based agonists and antagonists effectively suppress sex-hormone-driven signaling [82]. This leads to tumor regression or disease stabilization and justifies their widespread clinical use in prostate and breast cancer [83,87].

Beyond prostate cancer, BC provides another paradigm of hormone-dependent malignancy. Approximately 70% of all cases are hormone receptor-positive (HR+), defined by estrogen receptor (ER) expression and reliance on estrogen-driven transcriptional programs for proliferation and survival. ER exists in two main nuclear isoforms (ERα and Erβ) and a membrane-associated G-protein-coupled estrogen receptor (GPER). Aberrant activation of these receptors sustains tumor growth and contributes to therapy resistance. Conventional endocrine therapies exploit this dependency by blocking ER signaling. Selective estrogen receptor modulators (SERMs, e.g., tamoxifen) and estrogen receptor antagonists (e.g., fulvestrant) directly inhibit Erα activity, while aromatase inhibitors suppress estrogen synthesis, together forming the backbone therapy in HR+ breast cancer. Nevertheless, progesterone receptor antagonists such as mifepristone have also shown efficacy in specific BC subtypes [88]. Although these agents are not peptides, ongoing research has begun to explore peptide-based strategies to modulate ER pathways or deliver cytotoxic agents specifically to ER+ tumors. Examples include the synthetic peptide ERα17p, derived from the receptor itself, which exerts GPER-mediated antiproliferative activity in ER+ BC cells and xenograft models. Similarly, stapled peptides engineered to block ER–coactivator interactions disrupt ER signaling in vitro. Also, α-fetoprotein-derived peptides demonstrated tumor-suppressive activity in ER+ BC models [89,90,91,92]. The success of conventional endocrine therapy has prompted integration of targeted agents such as cyclin-dependent kinase 4 and 6 inhibitors (CDK4/6i), which significantly improve progression-free and overall survival [93]. However, resistance mechanisms—often mediated by compensatory pathways such as PI3K/AKT signaling—remain a major clinical challenge.

Beyond classical hormone receptor pathways, intracellular signaling complexes such as the NOD-like receptor protein 3 (NLRP3) inflammasome have emerged as important regulators of tumor biology. NLRP3 is activated in response to pathogen or damage-associated molecular patterns. Thus, it exerts pleiotropic effects in cancer progression, including promotion of epithelial–mesenchymal transition (EMT), angiogenesis, and modulation of pyroptotic cell death. Elevated NLRP3 expression in breast and other cancers has been correlated with an increase in tumor proliferation, enhanced migration, and chemoresistance [94]. Taken together, peptide-based hormone receptor modulators exemplify the clinical maturity and future potential of peptide therapeutics in oncology. Established GnRH agonists and antagonists already form the backbone of ADT in prostate cancer. Emerging peptide strategies targeting estrogen and progesterone receptor pathways highlight opportunities to expand treatment options in BC. Beyond classical endocrine signaling, novel targets such as the NLRP3 inflammasome further underscore the versatility of peptides as modulators of hormone-driven and resistance-associated pathways, reinforcing their role as a dynamic class of agents in precision cancer therapy.

### 3.2. Intracellular Protein–Protein Interactions

Following advances in hormone receptor and inflammasome targeting, peptide therapeutics are increasingly directed toward intracellular protein–protein interactions (PPIs), which constitute essential regulators of oncogenic signaling. PPIs are regulators of diverse cellular processes, and their dysregulation is frequently implicated in oncogenesis. Inhibitors of PPIs therefore constitute an important therapeutic class, designed to disrupt pathogenic protein complexes and restore cellular homeostasis. Small compounds have traditionally been explored for this purpose due to their ability to cross the plasma membrane and modulate intracellular targets. However, their limited capacity to block large or flat interaction surfaces and their inability to discriminate subtle structural changes—such as single-point mutations—restricts their long-term efficacy and often promotes drug resistance. By contrast, peptide-based inhibitors offer distinct advantages, including high surface complementarity, strong binding affinity, and improved selectivity for defined interaction motifs. Although the intracellular delivery of peptides remains a challenge, advances in peptide engineering (e.g., cell-penetrating motifs, stapled peptides, and chemical modifications) have expanded their potential as effective modulators of intracellular PPIs in cancer therapy [8]. Because of their ability to interfere with large and complex interaction surfaces, peptides are increasingly recognized as valuable tools for targeting PPIs that are difficult to modulate with conventional small molecules. In oncology, this strategy holds promise for overcoming therapeutic resistance and enhancing treatment precision. One example involves lactate dehydrogenase isoform 5 (LDH5), a key enzyme in aerobic glycolysis, frequently upregulated in tumors and correlates with aggressive phenotypes. A recent study demonstrated that peptide inhibitors can disrupt PPIs between LDHA subunits required for LDH5 tetramerization, thereby reducing its enzymatic activity. The rationally designed peptide cGmC9, with micromolar affinity for LDHA subunits, selectively inhibited LDH5 in cancer cells, impairing glycolytic metabolism [95].

H1P1R (Huntingtin-interaction protein 1-related), an endocytic adaptor protein, regulates actin assembly and clathrin-mediated endocytosis. In thyroid cancer, HIP1R promotes proliferation by mediating PTEN endocytosis, a critical tumor suppressor gene. Pharmacological inhibition of this interaction using flurbiprofen, a nonsteroidal anti-inflammatory drug with rapid onset and prolonged activity, was reported to block HIP1R-PTEN binding and attenuate tumor cell proliferation [96]. Recent studies revealed that HIP1R acts as a negative regulator of PD-L1, promoting its lysosomal degradation. Building on this mechanism, a chimeric peptide was engineered that combines the PD-L1-binding sequence of HIP1R with a lysosomal sorting signal. This design efficiently directs PD-L1 to lysosomal degradation, resulting in decreased PD-L1 expression and enhanced antitumor immunity in preclinical models [97].

The MTDH-SND1 interaction plays a pivotal role in BC progression, where metadherin (MTDH) and staphylococcal nuclease domain containing protein 1 (SND1) cooperate to promote oncogenic signaling. Recently, stabilized MTDH-derived disrupted this interaction, suppressing proliferation, invasion, and metastasis in triple-negative BC models. This is related with potential of inhibiting MTDH–SND1 binding and downregulating oncogenic signaling pathways such as NF-κB and PI3K/AKT [98,99]. In parallel, DCN1 (Defective in Cullin Neddylation 1) is frequently overexpressed in cancers including lung, head and neck, and cervical carcinomas, where it functions as an oncogene by enhancing neddylation of cullin-RING ligases. UBC12-mimetic peptides bind DCN1 with high affinity (K_d_ < 10 nM), thereby blocking the DCN1–UBC12 interaction and suppressing cullin1/3 neddylation. Functionally, this leads to impaired ubiquitin ligase activity, reduced tumor cell growth, and enhanced apoptosis [100]. Small molecules, such as WS-384, inhibit the DCN1-UBC12 interaction while simultaneously suppressing LSD1 activity. Although not peptide-based, WS-384 exemplifies the therapeutic relevance of disrupting oncogenic PPIs: it induces cell cycle arrest, DNA damage, and apoptosis through dual inhibition of LSD1 and DCN 1 pathways [99].

The chromatin-associated protein WD40 repeat domain 5 (WDR5) is a core component of the mixed-lineage leukemia (MLL) complex and plays an essential for epigenetic regulation. It contains two key binding sites: the WIN motif (WDR5–MLL interaction), a short peptide sequence, which recruits MLL1 and is critical for histone H3 lysine 4 (H3K4) methylation. Also, it contains the WDR5 biding motif (WBM) site, which mediates interactions with non-MLL partners like c-MYC. Through these interactions, WDR5 regulates gene expression programs linked to cell proliferation, differentiation and survival. Aberrant WDR5 contributes to oncogenesis in leukemia, breast, and prostate cancers [101]. Considerable efforts have focused on developing small-molecule inhibitors that target the WIN and WBM binding pockets to disrupt WDR5-mediated protein–protein interactions. While several of these compounds successfully impair WDR5 binding in vitro, their overall anticancer activity remains modest and in vivo efficacy is limited [101].

### 3.3. Cytotoxic Peptides

Cytotoxic peptides (CPs), whether naturally occurring or synthetically engineered, constitute a versatile class of bioactive molecules with promising antitumor activity. Their therapeutic relevance stems from their ability to interact with otherwise “undruggable” targets, including PPIs, while displaying minimal off-target effects. Importantly, the selectivity of CPs for tumor cells is largely determined by physiological and biochemical differences between malignant and healthy tissues [102]. Normal cell membranes are enriched in neutral phospholipids and sterols, with high cholesterol content that stabilizes membrane rigidity and protects against peptide insertion. In contrast, tumor cell membranes typically exhibit increase levels of anionic components, leading to an overall negative surface charge. This difference promotes preferential binding of cationic CPs, which can disrupt membrane integrity in a potential-dependent manner and exert broad-spectrum antitumor effects [8,102]. Mechanistically, CPs increase membrane permeability either by forming novel ion channels or by altering the function of existing ones, ultimately triggering cell death [103]. Building on these mechanisms, a variety of natural and synthetic CPs (e.g., melittin, magainin II, defesin, BMAP-28) have been investigated, as illustrated in the following examples.

**Figure 3:**
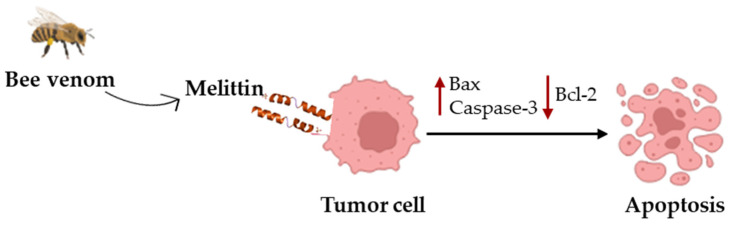
Cytotoxic peptide Melittin, obtained from bee venom, exerts anticancer activity inducing apoptosis by upregulating Bax and Caspase-3 and downregulating Bcl-2 (Created in BioRender.com https://app.biorender.com/illustrations/616ff88cd58e7800a5492543 (accessed on 16 December 2025)).

A well-known example of a cytotoxic peptide is melittin (GIGAVLKVLTTGLPALISWIKRKRQQ), a 26 amino acid amphipathic peptide derived from bee venom [37]. Its structure is characterized by a hydrophobic N-terminus and a hydrophilic amino acid located at the C-terminus, which facilitate robust interaction with lipid bilayers [104]. Melittin exerts anticancer activity through multiple mechanisms: it can induce apoptosis via upregulating Bax and Caspase-3 expression levels while downregulating the antiapoptotic Bcl-2 protein (Figure 3). Also, it also prevents angiogenesis, reduces inflammatory responses, and inhibits invasion and metastatic spread of tumor cells [104]. Preclinical studies confirm its broad antitumor activity across several malignancies (reviewed in [105]). For example, in breast cancer, it reduces viability and migration of MCF-7 and MDA-MB-231 cells, accompanied by apoptosis induction [106]. In hepatocellular carcinoma, this peptide inhibited proliferation of HepG2 and Huh7 cells by inducing apoptosis and restoring PTEN expression, while nano-liposomal formulations improved stability and in vivo antitumor efficacy [107,108,109]. Together, these studies highlight melittin’s potential as a free peptide and a component of nanocarrier formulations.

LL-37 (LLGDFFRKSKEKIGKEFKRIVQRIKDFLRNLVPRTES) is the only human cathelicidin protein, composed of 37 amino acids. LL-37 is characterized by a strong positive charge that favors interaction with the negatively charged membranes of many tumor cells. Functionally, the peptide is pleiotropic: depending on the cellular context, it can promote migration, proliferation, and invasion. On other hand, it regulates apoptosis and modulates immune responses [110,111]. This duality reflects its ability to engage distinct cell surface receptors, membrane channels, or intracellular pathways across different tumor types, leading to either protumorigenic or antitumor outcomes [110,111]. At high concentrations, LL-37 exhibits antitumor activity across several cancers, including colon, gastric, hematologic malignancies, and oral squamous cell carcinoma. For example, in pancreatic cancer, LL-37 suppresses suppressing autophagy and modulates the tumor immune microenvironment, resulting in reduced tumor growth in vitro and in vivo [111]. These findings highlight LL-37 as a dual-function peptide with potential for therapeutic exploitation, although its bidirectional roles demand careful context-specific evaluation before clinical application.

Magainin II (GIGKFLHSAKKFGKAFVGEIMNS) is a 23-amino-acid cationic peptide with an amphipathic *α*-helix structure, originally isolated from frog skin. It interacts with cell membranes by inserting into the phospholipid bilayer and forming ion-permeable pores [112]. In addition to its antimicrobial properties, the peptide has demonstrated anticancer activity in multiple tumor models. In human bladder cancer cell lines (RT4, 647V and 486P), it induces cytotoxic and antiproliferative efficacy by pore formation through membrane pore formation, while sparing normal human and murine fibroblasts. This reflects its preferential action on tumor-associated membrane compositions [113]. A similar dose-dependent cytotoxicity has been reported in BC, consistent with a membrane-disruptive mechanism [112]. The anticancer potential of magainin II and its analogues, such as pexiganan, has also motivated their incorporation into polymeric matrices to improve peptide stability and controlled released [114,115]. Although robustly validated in preclinical studies, clinical translation of Magainin-based therapeutics in oncology remains unproven.

Defensin-1 forms a structurally related but functionally diverse family present across plants and mammals. Plants defensins such as *Pisum sativum* defensin-1 (Psd1), *Medicago sativa* defensin-1 (MsDef1), and *Nicotiana alata* defensin-1 (NaD1) are typically ~30 amino acids. These peptides are stabilized by multiple disulfide bonds and display amphipathic properties that promote membrane association [116,117]. They exhibit direct cytotoxicity against tumor cells: Psd1 reduces the viability of B16-F10 mouse melanoma cells; MsDef1 selectively targets multidrug-resistant human breast cancer (MCF-7R) cells via dual engagement of glucosylceramide (GlcCer) and thioredoxin (Trx); and NaD1 induces necrosis-like death in multiple human cancer cell lines through binding to phosphatidylinositol 4,5-bisphosphate (PI(4,5)P_2_) at the plasma membrane [118,119]. Human β-defensin-1 (hBD-1) is often downregulated in cancer such as renal cell carcinoma and prostate). Restoring hBD-1 expression in malignant suppresses proliferation via interfering with growth factors signaling pathways (e.g., HER2). Additionally, it triggers apoptosis and endoplasmic reticulum stress, supporting it as therapeutic target and biomarker of disease progression [120,121,122,123,124].

Bovine myeloid antimicrobial peptide 28 (BMAP-28) is a cationic antimicrobial peptide from the cathelicidin family, isolated from bovine neutrophils, composed of 26 amino acids (GRFKRFRKKFKKLFKKLSKKKGGK) [125]. In membrane-mimetic environments, BMAP-28 adopts a strongly amphipathic α-helix conformation that confers preferential binding to the negatively charged surfaces of tumor cells. Upon membrane association, BMAP-28 compromises structural integrity and induces permeabilization, culminating in necrotic or apoptotic cell death [125]. In thyroid cancer models, it inhibits cell invasion and triggered apoptosis through activation of caspase-3 and caspase-9 in vitro, underscoring its potential as a targeted cytotoxic agent [125]. Although LL-37, magainin II, defensins, and BMAP-28 exemplify the diverse mechanisms through which cytotoxic peptides exert anticancer activity, their translational progress remains modest. The strongest evidence comes from membrane-active peptides such as magainin II and melittin analogues, which show consistent selectivity for negatively charged tumor membranes and potent cytolytic effects. However, context-dependent activity (as seen for LL-37), potential immunomodulatory effects, and risks of nonspecific membrane disruption limit safety margins. Stability, rapid proteolysis, and biodistribution challenges further restrict in vivo efficacy. While formulation within polymeric matrices or nanocarriers may mitigate some limitations, these approaches introduce new variables related to manufacturing, immunogenicity, and pharmacokinetics. Overall, cytotoxic peptides remain highly promising but require refined engineering, improved targeting strategies, and rigorous evaluation of toxicity and immunological effects to advance toward clinical application.

### 3.4. Cell-Penetrating Peptides as Therapeutics

Peptides that promote intracellular delivery of therapeutics molecules are broadly classified as cell-penetrating peptides (CPPs) or cell-targeting peptides (CTPs) [3]. CCPs are typically short, positively charged sequences that interact electrostatically with negatively charged components of the plasma membrane. This interaction enables them to cross cell barriers either by direct translocation or endocytosis [3]. Their mode of entry depends on several factors, including structural diversity, physiochemical properties, and the lipid compositions of the target cell membrane [3,4]. In contrast, CTPs recognize and bind to receptors that are overexpressed on the surface of specific cell types or tissues. This receptor-mediated recognition allows precise delivery of conjugated therapeutic payloads to disease cells. CPPs and CTPs can transport a broad diversity of cargo, proteins, nucleic acids, liposomes, and NPs. The transportation might occur through covalent conjugation, as in PDCs or through non-covalent association with peptide-engineered nanocarriers. However, challenges remain, including peptide stability, metabolic degradation, and potential off-target uptake. By combining high specificity, enhanced cellular uptake, and biocompatibility, CPPs and CTPs are emerging as versatile carriers for drugs, gene-editing tools, and imaging probes [3].

One of the best-characterized CPPs is TAT (YGRKKRRQRRR), an 11-amino-acid peptide originally derived from the trans-activator protein derived from HIV-1 [126]. When added exogenously to culture media, TAT rapidly enters cells, and at high concentrations it can directly cross membranes. In vitro studies using in vitro BBB models have shown that TAT is able to cross brain endothelial cell layers in a temperature-dependent manner, highlighting its potential as a shuttle for central nervous system delivery [126]. TAT has also been engineered into functional derivatives. A notable example is TAT-BECN1 peptide, generated by fusing TAT with a fragment of Beclin 1, a core component of Class III PI3K complexes that regulate autophagosome nucleation, maturation, endocytosis, and phagocytosis [127]. Tat-BECN1 can be delivered to cultured cells or isolated tissues or administered in vivo via intraperitoneal or intravenous injection, or infusion into animals, provided it is in aqueous solution. Once internalized, it targets specific organelles for perinuclear delivery and induces lysosomal damage and cell death in BC cells [118].

Another major class of delivery peptides is the angiopep family of peptides derived from the Kunitz domain of human aprotinin. These peptides have a notable capacity for central nervous system delivery (e.g., targeting glioblastoma and brain metastases of breast cancers) [128]. Among them, angiopep-2 (ANG-2) is the most widely used member of this family. This 19-amino-acid peptide (TFFYGGSRGKRNNFKTEEY) exhibits high transcytosis efficiency, broad brain volume of distribution, and specific interaction with low-density lipoprotein receptor-related protein 1 (LPR-1) [128]. ANG-2 has been extensively investigated as a brain-targeting ligand for glioblastoma and breast cancer brain metastases [128]. One of best-known derivatives is ANG1005, a PDC in which three paclitaxel molecules covalently linked to ANG-2. This construction has demonstrated improved penetration across BBB and enhanced tumor accumulation for breast cancers [129]. Structural studies of ANG-2 binding to LPR-1 moieties (CR56 and CR17) showed that distinct ligand-binding domains contribute differently to its transport: CR56 provides site-specific interaction crucial for transcytosis, whereas CR17 mediates less specific binding but supports apolipoprotein internalization under physiological conditions [128]. A phase II clinical trial (NCT01967810) demonstrated that a dose of 600 mg/m^2^ was safe but the primary efficacy endpoint (i.e., objective response rate (ORR) and progression-free survival (PFS)) were not met. Thus, no further studies were conducted in glioma patients with this peptide [130].

CPPs and CTPs illustrate two complementary yet functionally distinct strategies for intracellular delivery, and their comparative performance depends largely on the therapeutic context. CPPs such as TAT offer unmatched versatility and strong internalization capacity across diverse cell types, making them ideal for applications requiring broad uptake or cytosolic delivery of macromolecular cargo. However, their lack of intrinsic specificity increases the risk of off-target accumulation, and their rapid endosomal sequestration often limits effective cytosolic release. In contrast, CTPs such as the angiopep family—particularly ANG-2—demonstrate superior targeting fidelity, leveraging receptor-mediated transcytosis to achieve efficient tissue-selective penetration, most notably across the BBB. ANG-2-based constructs, such as ANG1005, consistently outperform classical CPPs in brain delivery applications, exhibiting higher brain parenchymal distribution and more predictable pharmacokinetics. Nonetheless, their efficiency is tightly linked to receptor expression profiles and may vary across tumor subtypes or disease stages. Overall, TAT exemplifies maximal delivery breadth but limited specificity, whereas ANG-2 represents precision targeting with more restricted applicability. These contrasting properties underscore that no single peptide modality is universally superior. Instead, delivery performance hinges on the interplay between peptide structure, receptor availability, and the biological barriers associated with each cancer indication.

Considering the different class of peptides discussed in this section, it is feasible to state that each class offers unique strengths but also faces distinct translational constraints. Cytotoxic peptides provide potent membrane-disruptive activity yet are limited by systemic toxicity and short half-life. PDCs improve precision through modular design but depend heavily on linker stability and receptor density for efficacy. Intracellular PPI-targeting peptides excel at disrupting complex interaction surfaces that small molecules cannot reach, though their therapeutic success hinges on improved intracellular delivery systems. Immune-checkpoint-modulating peptides promise lower cost and deeper tissue penetration than antibodies but remain in early development due to concerns about in vivo stability and sustained receptor occupancy. CPPs and CTPs expand delivery possibilities, with CPPs offering breadth of uptake and CTPs providing exquisite specificity. Nevertheless, both require careful optimization to balance internalization efficiency against off-target accumulation. Thus, these classes exemplify the versatility of peptide therapeutics while highlighting that no single strategy is universally optimal. Instead, their effectiveness depends on matching each modality to the biological barrier, molecular target, and disease context most suited to its strengths.

## 4. Peptide-Based Immune Modulation

Peptide-based immune modulation represents a rapidly expanding area in cancer therapy, leveraging the ability of short amino acid sequences to reshape both innate and adaptive immune responses. Unlike delivery-focused peptides, immunomodulatory peptides act directly on immune cells or signaling pathways, enhancing antigen presentation, activating cytotoxic lymphocytes, suppressing immunosuppressive networks, or modulating cytokine release within the tumor microenvironment [131]. Their features make them attractive candidates for next-generation immunotherapies, including vaccines, immune-activating adjuvants, checkpoint-enhancing peptides, and agents that reverse tumor-induced immune dysfunction [132]. Within this landscape, peptide-based vaccines stand out as one of the most clinically advanced applications, offering a targeted and adaptable approach to stimulate antitumor immunity.

### 4.1. Therapeutic Peptides as Anticancer Vaccines

Vaccine development remains a global health priority, driven not only by persistent viral threats but also by the growing need for effective cancer immunotherapies. In oncology, peptide-based vaccines have gained attention for their ability to elicit targeted antitumor immune responses while offering manufacturing simplicity, high safety, and adaptability [133]. Unlike conventional vaccines that rely on whole cells or viral vectors, peptide vaccines rely on short antigenic fragments derived from tumor-associated or tumor-specific proteins. These fragments can be synthesized with precision, modified to enhance immunogenicity and specificity, providing a flexible platform that can be rapidly adapted to tumor heterogeneity and antigenic variation [134,135]. From an immunogenicity perspective, peptide-based vaccines present defined epitopes at high density, improving antigen recognition and enhancing adjuvant efficacy [134,135]. Their mechanism relies on training the immune system to recognize either tumor-associated antigens (TAAs) or tumor-specific antigens (TSAs), facilitating precise immune targeting of malignant cells. TAAs are normally expressed at low levels in healthy tissues but are overexpressed in tumors, often contributing to cell cycle regulation or survival pathways. By contrast, TSAs arise from oncogenic driver mutations that generate novel peptide sequences absent from normal tissues, making them highly immunogenic and less likely to provoke tolerance. This distinction has made TSA-based vaccines particularly attractive targets in precision oncology [136].

Peptide vaccines typically consist of short sequences (8–12 amino acids) presented on human leukocyte antigen (HLA) molecules and capable of stimulating by CD4^+^ helper T cells and CD8^+^ cytotoxic T cells [136]. They can induce active de novo immune responses or augment the activity of other passive immunotherapies such as checkpoint inhibitors, where they help sustain or reactivate T cell activity. Numerous clinical trials have demonstrated that peptide vaccines are safe, stable, inexpensive to manufacture, and capable to generate tumor-specific T cell responses [136]. Unlike chemotherapy, they lack systemic toxicity, have low carcinogenic potential, and are resistant to pathogen contamination. Nonetheless, challenges remain. Peptide antigens alone are usually weakly immunogenic due to rapid degradation at the injection site and the absence of costimulatory signals. Thus, the selection of suitable adjuvants is essential. For example, Montanide ISA-51, a water-in-oil emulsion, is frequently used in clinical trials. By forming a depot at the injection site, it prolongs antigen availability, enhances epitope presentation, and promotes sustained T cell activation. Clinical trials in lung cancer and melanoma using Montanide-formulated peptide vaccines report improved immune activation, though variability across patients remains considerable [136,137]. Several peptide-based vaccines have advanced to clinical testing, highlighting their potential as safe and immunogenic cancer therapies. Table 4 depicts some examples, including their target antigen, mechanism of action, and cancer type.

One of the most extensively studied vaccines is IMA901, a multiple tumor-associated peptide (TUMAP) vaccine for renal cell cancer treatment [138]. In a phase I/II clinical trial, IMA901 induced robust T cell-mediated immune responses against multiple tumor antigens and excellent safety profile with no treatment-related adverse effects [138]. These results provided early proof-of-concept for the feasibility of multi-epitope peptide vaccines in oncology and encouraged further investigation of similar peptide-based vaccine platforms.

NeuVax is a single-peptide vaccine, specifically the class I peptide E75, developed to target HER2-positive breast cancer. HER2 is a well-validated antigen in oncology, overexpressed in a subset of BCs, making it an attractive target for vaccine strategies. The E75 peptide stimulates CD8+ T cells to recognize and kill HER2-expressing tumor cells. In clinical trials, it has been delivered in combination with granulocyte-macrophage colony-stimulating factors (GM-CSF) as an adjuvant in phase II/III clinical trials. Patients in those trials received either NeuVax plus GM-CSF or GM-CSF alone in early-stage BC and exhibited durable immune responses, likely enhanced by the GM-CSF component [139].

Another peptide vaccine for BC treatment is GP2 (IISAVVGIL), also derived from HER2. Preclinical studies showed GP2 is immunogenic, and subsequent phase I trials demonstrated its safety and ability to elicit peptide-specific immune responses in patients [140]. In a phase II trial (NCT00524277), GP2 + GM-CSF was evaluated as adjuvant therapy in BC patients with any level of HER2 expression (IHC 1–3+). The results confirmed good safety, and subset analyses suggested favorable disease-free survival (DFS) in HER2-positive patients, with estimated 5-year DFS of 94% in vaccinated versus 89% in controls (per-treatment analysis: 100% vs. 89%, *p* = 0.08) [141,142,143]. Importantly, GP2 has also been tested in combination with trastuzumab (Herceptin) in a phase I trial, showing that co-administration is safe and immunogenic and may promote epitope spreading (i.e., widening of the antitumor immune response) [144]. A phase II multi-center trial comparing GP2 + GM-CSF versus GM-CSF alone observed that GP2 and AE37 vaccines were safe, with limited toxicity linked primarily to GM-CSF. In specific subgroups, there was suggestion of improved DFS with vaccination [142,145]. Moreover, the ongoing phase III trial Flamingo-01 (GLSI-100 / GP2 + GM-CSF) is now recruiting HER2/neu-positive, HLA-A2 positive patients to more robustly test the efficacy and safety of the GP2 vaccine in adjuvant settings [146].

GV1001, derived from a human telomerase reverse transcriptase (hTERT) sequence (residues 611–626: EARPALLTSRLRFIPK), has been evaluated in multiple cancer types including prostate cancer, non-small cell lung cancer (NSCLC), melanoma, and pancreatic cancer [147]. In a phase II trial in NSCLC (CTN-2006), 16 out of 20 patients developed a GV1001-specific immune response, and those responders had a median progression-free survival (PFS) of 371 days versus 182 days for non-responders (though the difference was underpowered) [148]. In earlier trials (CTN-2000), 11/24 patients mounted a GV1001 immune response, and immune responders showed improved survival compared to non-responders (median survival ~19 months vs. ~3.5 months) [148,149]. Beyond its vaccine role, GV1001 has demonstrated direct anticancer and antiangiogenic properties. Preclinical studies demonstrated that GV1001 inhibits endothelial cell proliferation, migration, invasion, tube formation, and microvessel sprouting by suppressing VEGF-A/VEGFR-2 signaling and downregulating matrix metalloproteinase-2 (MMP-2) expression. In cancer cell lines (e.g., gastric, colorectal models), GV1001 reduces viability and induces apoptosis [150]. In NSCLC cell models, this peptide also reduced VEGF secretion and cell invasiveness. In mouse xenograft models, treatment resulted in decreased tumor growth, increased apoptosis, and reduced microvessel density in tumors, supporting its dual immunologic and direct cytotoxic/antiangiogenic activity [150,151]. Additionally, GV1001 has non-canonical roles related to tissue protection and modulation of fibrotic responses. In irradiated human keratinocytes and fibroblasts, GV1001 suppressed TGF-β/Smad signaling, reduced fibrotic gene expression (e.g., collagens, fibronectin), and mitigated epithelial–mesenchymal transition (EMT) phenotypes, suggesting radioprotective and antifibrotic properties [152]. In clinical practice, trials combining GV1001 with chemotherapy (e.g., gemcitabine/capecitabine in pancreatic cancer) were tolerable but did not significantly improve survival outcomes [153,154]. In BC, retrospective data from 63 patients receiving GV1001 plus chemotherapy showed a disease control rate between ~50–66% depending on subtype, with modest survival outcomes and improved quality of life, and no significant additional toxicity beyond chemotherapy’s own side effects [154]. Taken together, GV1001 exemplifies a hybrid peptide vaccine—combining immunogenic stimulation with direct biological activity—and underscores the promise and the challenges of translating peptide vaccines into effective cancer therapies.

Collectively, peptide-based cancer vaccines such as IMA901, NeuVax, GP2, and GV1001 exemplify the versatility and safety of peptide-based immunotherapies. They can elicit targeted T cell responses and complement conventional therapies. Nevertheless, several challenges remain. These include improving peptide immunogenicity, optimizing adjuvant formulations, and refining delivery strategies that overcome rapid degradation and tumor-induced immunosuppression. Continued innovation in these areas will be essential for translating peptide-based vaccines into broadly effective anticancer therapies.

### 4.2. Immune Checkpoint Inhibitors

Immune checkpoint inhibitors (ICIs) represent one of the most transformative advances in modern oncology. Tumors exploit immune-regulatory pathways to evade immune surveillance, and ICIs act by blocking these inhibitory signals, thereby restoring T cell cytotoxicity. Over the past decade, they have reshaped the standard of care for solid tumors and hematologic malignancies. Their therapeutic effects stem from preventing inhibitory receptors on T cells from engaging with their ligands in the tumor microenvironment (TME), ultimately restoring cytotoxic activity and enhancing antitumor immunity [155]. Key checkpoints include cytotoxic T lymphocyte-associated antigen 4 (CTLA-4), programmed cell death protein 1 (PD-1), and its ligand PD-L1, and more recently LAG-3 and T cell immunoglobulin and mucin-domain containing-3 (TIM-3). Despite their clinical success, ICIs remain associated with immune-related adverse events (irAEs), reflecting systemic immune activation and off-target effects [155,156].

**Figure 4:**
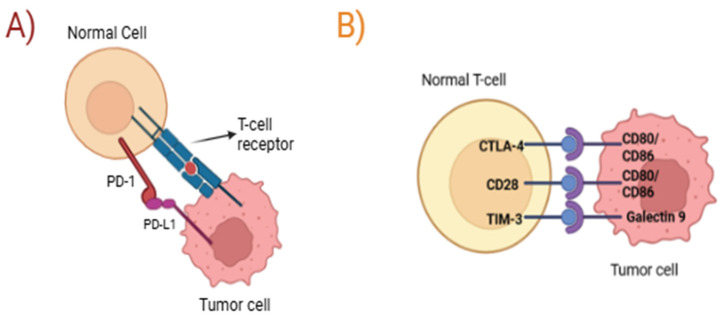
Immune checkpoint interactions between a normal T cell and a tumor cell. (**A**) depicts PD-1 receptor on a normal cell binding to PD-L1 on tumor cells. (**B**) represents CTLA-4 and CD28 on T cells interacting with CD80/CD86 on tumor cells and TIM-3 interact with galectin-9 on tumor cells, influencing T cell activation. Created in BioRender.com. https://app.biorender.com/illustrations/68666472544565ba1be93850 (accessed on 16 December 2025) and adapted from [147].

Checkpoint pathways regulate immunity at distinct stages. CTLA-4 competes with the co-stimulatory receptor CD28 for binding to the B7 family ligands (B7-1/CD80 and B7-2/CD86) expressed on antigen-presenting cells (APCs). This reduces the activation of naïve T cells and limits sustained stimulation in inflamed tissues [155,156]. PD-1, expressed on activated T cells, recruits phosphatase SHP-2 upon engagement with PD-L1 or PD-L2, suppressing TCR and CD28 signaling [155]. Among these ligands, PD-L1 expression is particularly relevant in oncology, as it is strongly upregulated in normal and malignant tissues in response to inflammatory signals such as interferon-gamma (IFNγ) secreted by activated T cells. In addition, LAG-3, expressed on activated CD4^+^ and CD8^+^ T cells, contributes to T cell exhaustion in TME, while TIM-3 interacts with ligands including galectin-9 (Gal-9) to dampen effector activity [156]. Under physiological conditions, T cell activation requires recognition of tumor antigens presented on major histocompatibility complex (MHC) molecules, which triggers clonal expansion and infiltration into the tumor, followed by cytotoxic elimination of antigen-expressing cancer cells [157]. Checkpoint signaling disrupts these processes and restrains effective antitumor responses (Figure 4).

Monoclonal antibodies (e.g., pembrolizumab, nivolumab, cemiplimab, atezolizumab, durvalumab, etc.) were the first clinically approved ICIs which validated PD-1, PD-L1, and CTLA4 as therapeutic targets and transformed cancer management [155,156,157,158,159,160,161,162,163]. However, antibody-based ICIs present limitations, such as high production costs, intravenous administration, and systemic toxicities. These constraints have motivated the development of peptide-based checkpoint inhibitors, which offer potential benefits in stability, manufacturability, and tissue penetration. Table 5 summarizes some representative peptides that inhibit immune checkpoints and are relevant to cancer treatment.

Several peptide inhibitors targeting PD-1/PD-L1 have shown promising results in early studies. CLP002 (WHRSYYTWNLNT) binds PD-L1 with high affinity and blocks its interaction with CD80, restoring the proliferation and survival of tumor-infiltrating T cells. Additionally, it prevents their apoptosis and thereby reactivating antitumor immunity [159]. YT-16 (YRCMISYGGADYKCIT), a cyclic peptide identified by computational screening, enhances T cell cytokine secretion and cytotoxicity [160]. Another example is AUNP-12 (LKEKKLGEFGKAKGLGKDGK), a synthetic peptide designed as a competitive antagonist targeting PD-L1, leveraging extracellular domain of human PD-1 [162]. By binding with high affinity to PD-L1, AUNP-12 disrupts the PD-1/PD-L1 interaction, re- activation T cell responses and exerting antitumor effects. Beyond its therapeutic potential, AUNP-12 has also been incorporated into near-infrared fluorescence molecular imaging probes to noninvasively monitor PD-L1 expression in vivo, offering a tool to stratify patients and evaluate immune responses during therapy [162].

In addition, peptides targeting alternative inhibitory receptors are also emerging. One example is P26 peptide (GLIPLTTMHIGK), which targets TIM-3, an inhibitory receptor of *TIM* gene family located on human chromosome 5. TIM-3 is implicated in immune suppression through interactions with ligands such as Gal-9 and high mobility group protein 1 (HMGB1). P26 competes with Gal-9 for TIM-3 binding, thereby blocking inhibitory signaling. In preclinical models, P26 restored T cell function and exerted antitumor effects in vivo [161]. Although such approaches remain earlier in development, they highlight the value of extending peptide-based strategies beyond PD-1/PD-L1.

For CTL-4, peptide inhibitors are less advanced but represent a promising complementary strategy. One example is p334 peptide (ARHPSWYRPFEGCG), a 14-amino-acid sequence with hydrophilic properties that mimics the loop region of CTLA-4 responsible for binding to the B7 receptor. By competitively interfering with this interaction, p334 acts as a peptide antagonist of CTLA-4, offering a promising alternative approach for immune checkpoint blockade [163].

Taken together, these examples highlight the expanding potential of peptide-based ICIs. Peptides that target PD-1/PD-L1 are closest to advancing to translational applications, whereas CTLA-4 and TIM-3 peptide inhibitors remain at earlier stages. Their modularity, tissue penetration, and manufacturability suggest that peptides could help overcome some limitations of monoclonal antibodies and broaden therapeutic options in cancer immunotherapy.

## 5. Translation Challenges and Clinical Limitations

Despite important advances in peptide design and delivery, the clinical translation of peptide therapeutics in oncology continues to face predictable and interrelated challenges. Together, these factors explain why many peptides with strong preclinical activity fail to progress beyond early-phase trials [164]. Below, we summarize the key issues, explain their importance for different peptide modalities, and highlight where progress is being made.

A central challenge arises from pharmacokinetic instability. Peptides are typically small and hydrophilic, which favors rapid renal clearance and short plasma half-lives. They are also susceptible to proteolysis by circulating and tumor-associated peptidases. The combined result is often inadequate systemic exposure and variable biodistribution [165]. In response, multiple chemical strategies—cyclization, D-amino acid substitution, PEGylation, lipidation, or albumin-binding motifs—have been developed to improve peptide stability. However, these modifications may change receptor binding, tissue distribution or immunogenicity, and complicate regulatory assessment. Thus, stability can be engineered, but not without trade-offs [166].

These pharmacokinetic barriers closely intersect with challenges in formulation and delivery. Efficient delivery to the intended intratumoral compartment remains the major practical barrier. Unmodified peptides exhibit low oral bioavailability, limited ability to cross biological barriers such as the BBB, and heterogeneous distribution within solid tumors [167,168]. To overcome these obstacles, diverse delivery systems have been explored, including PDCs, CPP-mediated uptake, nanoparticle encapsulation, and controlled-release depots. Yet, each approach brings its own limitations: unstable linkers in PDCs, non-specific uptake in CPPs, hepatic and splenic sequestration in NPs, or local inflammation with depot-forming adjuvants. Importantly, these multi-component systems also raise issues of scalability and batch-to-batch reproducibility, complicating clinical deployment.

Manufacturing peptide therapeutics at clinical grade demands high purity (>95%), rigorous removal of synthesis by-products, and confirmation of structural fidelity (including disulfide bond architecture, stereochemistry, and post-synthetic modifications) [169]. Long, cysteine-rich, or highly hydrophobic peptides increase synthesis complexity and cost. Multi-component constructs (PDCs, peptide–NP hybrids, vaccine + adjuvant systems) need multi-step Good Manufacturing Practice (GMP) manufacturing and complex quality control to ensure batch comparability. This complicating dossier preparation, quality control expectations, and comparability assessments across batches. Furthermore, regulatory agencies increasingly require immunogenicity testing, even for small peptides, particularly in chronic dosing regimens [170,171]. These factors lengthen timelines and add development costs.

Across modalities there are recurring translational patterns. Peptide vaccines reliably generate antigen-specific T cell responses, but clinical benefit is often limited when used as a monotherapy because of low intrinsic immunogenicity, HLA restriction, and tumor immune evasion. PDCs can demonstrate target engagement and improved tissue delivery (e.g., ANG1005/GRN1005 for BBB crossing), yet dose-limiting toxicities, complex PK, and suboptimal payload release frequently curb efficacy. Membrane-active cytotoxic peptides show potent preclinical activity but are hampered by systemic toxicity in vivo unless tightly targeted or formulated. Immune-modulating and checkpoint-targeting peptides remain promising but need robust evidence of sustained receptor occupancy and favorable PK to rival antibodies. The proximate cause of many failures is not the lack of a biological mechanism but an inability to achieve the right exposure/target engagement at the tumor while keeping off-target effects manageable. Immune checkpoint-modulating peptides targeting PD-1/PD-L1, TIM-3, and CTLA-4 are at early developmental stages. While they offer theoretical advantages over antibodies, better tissue penetration, lower cost, and modularity, their in vivo stability and ability to sustain receptor occupancy remain insufficiently validated. Across these platforms, a recurring theme emerges: translation fails when pharmacokinetic and formulation challenges outweigh biological potency. This gap between molecular promise and clinical performance underscores the need for improved delivery technologies, rational peptide engineering, and more predictive preclinical models.

Despite these barriers, the field is progressing rapidly. Innovation in computational design, backbone engineering, next-generation linkers, and stimuli-responsive delivery systems. At the same time, advances in regulatory frameworks for complex biologics and long-acting peptides are also beginning to streamline translation, reducing development bottlenecks and improving clinical feasibility [164]. Importantly, these technological gains coincide with a broader conceptual shift: therapeutic peptides are no longer viewed as ancillary molecules but as central components of precision oncology.

Therapeutic peptides offer a unique combination of molecular specificity, biocompatibility, and controlled biodegradability. Their versatility—spanning drug targeting, intracellular inhibition, immune activation, and tumor penetration—positions them as valuable complements or alternatives to established small molecules and biologics. Advances in peptide vaccines, PDCs, CPP-based delivery systems, and intracellular PPI inhibitors continue to broaden their therapeutic scope. Modern synthetic chemistry now allows rational optimization of receptor affinity, protease resistance, and membrane permeability, while engineered nanoparticles, liposomes, and polymeric carriers increasingly address challenges of bioavailability and tumor heterogeneity. Nonetheless, several priorities remain critical for clinical maturation: extending peptide half-life, ensuring controlled release at the tumor site, improving delivery to deep or heterogeneous tissues, and sustaining immune activation over time. Combinatorial approaches with immunotherapy, radiotherapy, targeted small molecules, or chemotherapeutics are likely to maximize clinical benefits. Preventive or early-stage disease applications, particularly via peptide vaccines, also merit exploration.

In conclusion, therapeutic peptides are emerging not merely as adjunct agents but as foundational elements of next-generation cancer therapies. A summary of representative peptide candidates currently in clinical trials is provided in Table 6, highlighting the breadth of ongoing development. Additionally, the table shows that despite challenges remain, they are increasingly addressable through modern biotechnology and rational therapeutic design.

Together, the diversity of targets, modalities, and clinical outcomes highlights the substantial progress already made. The success will depend on integrated development where pharmacology, delivery technology and regulatory strategy are co-designed from the outset.

## Figures and Tables

**Table 1:** Tumor-homing peptides used for guided delivery of exosomes to tumor cells.

THP	Sequence	Target	Target Disease	Refs.
iRGD	CRGDCKGDC	αvβ3 integrin	Breast Cancer, Carcinoma	[24,29]
tLyP-1	CGNKRTRGC	p32	Breast Cancer	[24]
GE11	YHWYGYTPQNVI	EGFR	Lung Cancer	[30]
T7	HAIYPRH	TfR	Glioblastoma	[31]

**Table 2:** Examples of the peptide-targeted radionuclides.

Peptide	Radionuclide	Target	Cancer Type	Refs.
FAP-2286	^177^Lu	Fibroblast activation protein	Pancreatic, ovarian and colorectal cancer	[44]
PSMA-targeted peptide	^177^Lu	Prostate-specific membrane antigen	Prostate cancer	[45]
Various peptides	^90^Y	Tumor-specific receptors	Colorectal cancer and liver metastases	[46]
Octreotide	^90^Y, ^177^Lu	Somatostatin receptor subtype 2	Neuroendocrine neoplasms and GH-secreting tumors	[47,48,49,50]

**Table 3:** Comparison of peptide-based GnRH agonists and antagonists used in hormone-dependent cancers.

Class	Examples (Peptide Drugs)	Mechanism of Action	Clinical Features	Advantages	Limitations
GnRH Agonists	Leuprolide, Goserelin, Triptorelin, Buserelin	Initially stimulate pituitary GnRH receptors → transient surge in LH, FSH, testosterone (flare) → receptor desensitization with continuous use → suppression of gonadotropins and androgens	Widely used in prostate and premenopausal breast cancer	Long clinical track record; effective androgen suppression; various formulations (injections, implants)	Tumor flare effect (requires antiandrogen co-therapy); slower onset of castration
GnRH Antagonists	Degarelix, Cetrorelix, Relugolix (oral, non-peptide)	Competitively block GnRH receptors at pituitary → immediate suppression of LH/FSH → rapid fall in testosterone without flare	Increasing use in advanced/metastatic prostate cancer; relugolix shown safer in cardiovascular risk patients	Immediate effect; avoids tumor flare; oral option (relugolix); favorable safety profile	Higher cost (in some cases); shorter history of clinical use compared to agonists

**Table 4:** Examples of peptides as anticancer vaccines and their mechanism of action.

Peptide Vaccine	Target Antigen	Mechanism of Action	Cancer Type	Refs.
IMA901	Multiple TUMAPs	Induces T cell response	Renal cell carcinoma	[138]
NeuVax (E75)	HER2	Stimulates CD8+ T cells via GM-CSF	Breast cancer	[139]
GP2	Her2	Induces HER2-specific CD8+ T cells	Breast cancer	[140,141,142,143,144,145,146]
GV1001	hTERT	Penetrates cells, inhibiting proliferation and inflammation	Prostate and pancreatic cancer and melanoma	[135,136,137,138]

**Table 5:** Examples of peptides that inhibit immune checkpoints.

Checkpoint Target	Peptide (Sequence)	Mechanism of Action	Key Findings/Outcomes	Ref.
PD-L1	CLP002 (WHRSYYTWNLNT)	Binds PD-L1 with high affinity, blocks PD-L1/CD80 interaction	Restores T cell proliferation and survival; prevents apoptosis of tumor-infiltrating T cells	[159]
PD-L1	AUNP-12 (LKEKKLGEFGKAKGLGKDGK)	Competitive antagonist derived from PD-1 extracellular domain	Blocks PD-1/PD-L1; restores T cell activation; also used in near-infrared imaging probes for PD-L1 monitoring in vivo	[162]
PD-L1/PD-1	YT-16 (YRCMISYGGADYKCIT)	Cyclic peptide antagonist identified via virtual screening	Enhances T cell cytokine secretion and cytotoxicity	[160]
TIM-3	P26 (GLIPLTTMHIGK)	Competes with Galectin-9 for TIM-3 binding	Restores T cell function; in vivo antitumor activity	[161]
CTLA-4	p334 (ARHPSWYRPFEGCG)	Mimics CTLA-4 loop region binding to B7 ligands	Blocks CTLA-4/B7 interaction; potential CTLA-4 antagonist	[163]

**Table 6:** Representative ongoing clinical trials evaluating peptide-based therapeutics across different modalities.

Peptide/ Modality	Target	Payload/ Adjuvant	Indication	Trial Phase	Identifier (NCT)	Outcome/Status (Summary)
ANG1005 (GRN1005)—PDC (angiopep-2–paclitaxel)	LRP1 (LRP-1 mediated transcytosis)	3 × paclitaxel (conjugate)	Brain metastases/glioma	I/II → III program	NCT03613181	Demonstrated BBB penetration and signals of activity in early trials; progressed to larger trials exploring brain mets/leptomeningeal disease. Evidence supports BBB transport but mixed efficacy signals; safety manageable with hematologic DLTs reported.
GP2 (vaccine) FLAMINGO-01	HER2 (E75 family peptide fragment)	GM-CSF adjuvant	HER2 + BC (adjuvant therapy)	Phase III	NCT05232916	Designed to assess disease-free survival in adjuvant patient subset; prior phase I/II data supportive of immunogenicity; phase III active.
NeuVax/(E75) peptide vaccine	HER2 (E75)	GM-CSF adjuvant	Early-stage HER2+ BC	Phase II/III	NCT01479244 (ongoing)	Large phase trials produced mixed/limited efficacy signals; some arms terminated or re-focused (important lesson on HLA restriction and adjuvant dependence).
GV1001 telomerase peptide vaccine	hTERT	Various adjuvant regimens (clinical trials varied)	NSCLC, pancreatic cancer, melanoma (multiple trials)	Phase I/II	NCT03184467 (example recent trial)	Induces immune responses; mixed clinical efficacy in larger trials; some positive signals in immune responders but overall inconsistent survival benefit.
^177^Lu -FAP-2286 peptide-guided radionuclide	Fibroblast activation protein (FAP)	^177^Lu radioligand payload	Various advanced solid tumors with FAP expression	Early clinical (first-in-human/expansion cohorts)	NCT04939610 (and related early-phase studies)	Shows selective tumor uptake; preliminary antitumor activity reported in small cohorts; further trials underway to define efficacy/toxicity profile.
Selected PRRT examples (benchmark) somatostatin analogues	SSTR2	^177^Lu, ^90^Y, ^111^In labeled analogues	Neuroendocrine tumors (NETs)	Established clinical use (approved regimens)	many NCTs/registries (e.g., Lutetium-^177^DOTATATE programs)	Strongest clinical evidence among peptide modalities—durable disease control in selected NET patients; established safety profile and regulatory approvals.

## Data Availability

The original contributions presented in this study are included in the article. Further inquiries can be directed to the corresponding author.

## References

[1] Marqus S., Pirogova E., Piva T.J. (2017). Evaluation of the use of therapeutic peptides for cancer treatment. J. Biomed. Sci..

[2] https://www.cancer.gov/about-cancer/understanding/what-is-cancer.

[3] Moreno-Vargas L.M., Prada-Gracia D. (2024). Cancer-Targeting Applications of Cell-Penetrating Peptides. Int. J. Mol. Sci..

[4] Yavari B., Mahjub R., Saidijam M., Raigani M., Soleimani M. (2018). The Potential Use of Peptides in Cancer Treatment. Curr. Protein Pept. Sci..

[5] Fosgerau K., Hoffmann T. (2015). Peptide Therapeutics: Current Status and Future Directions. Drug Discov. Today.

[6] FDA Guidances. https://www.fda.gov/drugs/guidance-compliance-regulatory-information/guidances-drugs.

[7] Bose D., Roy L., Chatterjee S. (2022). Peptide therapeutics in the management of metastatic cancers. RSC Adv..

[8] Vadevoo S.M.P., Gurung S., Lee H.S., Gunassekaran G.R., Lee S.-M., Yoon J.-W., Lee Y.-K., Lee B. (2023). Peptides as Multifunctional Players in Cancer Therapy. Exp. Mol. Med..

[9] Milewska S., Sadowska A., Stefaniuk N., Misztalewska-Turkowicz I., Wilczewska A.Z., Car H., Niemirowicz-Laskowska K. (2024). Tumor-Homing Peptides as Crucial Component of Magnetic-Based Delivery Systems: Recent Developments and Pharmacoeconomical Perspective. Int. J. Mol. Sci..

[10] Thongpon P., Tang M., Cong Z. (2025). Peptide-Based Nanoparticle for Tumor Therapy. Biomedicines.

[11] Omidian H., Cubeddu L.X., Wilson R.L. (2025). Peptide-Functionalized Nanomedicine: Advancements in Drug Delivery, Diagnostics, and Biomedical Applications. Molecules.

[12] Jadhav K., Abhang A., Kole E.B., Gadade D., Dusane A., Iyer A., Sharma A., Rout S.K., Gholap A.D., Naik J. (2025). Peptide-Drug Conjugates as Next-Generation Therapeutics: Exploring the Potential and Clinical Progress. Bioengineering.

[13] Sagar B., Gupta S., Verma S.K., Reddy Y.V.M., Shukla S. (2025). Navigating cancer therapy: Harnessing the power of peptide-drug conjugates as precision delivery vehicles. Eur. J. Med. Chem..

[14] Xiao W., Jiang W., Chen Z., Huang Y., Mao J., Zheng W., Hu Y., Shi J. (2025). Advance in peptide-based drug development: Delivery platforms, therapeutics and vaccines. Signal Transduct. Target. Ther..

[15] Liu M., Fang X., Yang Y., Wang C. (2021). Peptide-Enabled Targeted Delivery Systems for Therapeutic Applications. Front. Bioeng. Biotech..

[16] Tran N.H., Nguyen D.D., Nguyen N.M., Tran C., Thi N.T.N., Ho D.T., Nguyen H.-N., Tu L.N. (2023). Dual-targeting exosomes for improved drug delivery in breast cancer. Nanomedicine.

[17] Huang C.-C., Liu F.-R., Feng Q., Pan X.-P., Song S.-L., Yang J.-L. (2021). RGD4C Peptide Mediates Anti-p21Ras scFv Entry into Tumor Cells and Produces an Inhibitory Effect on the Human Colon Cancer Cell Line SW480. BMC Cancer.

[18] Yuan D., Lu Z., Xu X., Liu W. (2024). RGD Peptide-Conjugated Polydopamine Nanoparticles Loaded with Doxorubicin for Combined Chemotherapy and Photothermal Therapy in Thyroid Cancer. Discov. Oncol..

[19] Wang B., Tang D., Cui J., Jiang H., Yu J., Guo Z. (2024). RGD-Based Self-Assembling Nanodrugs for Improved Tumor Therapy. Front. Pharmacol..

[20] Sheikh A., Alhakamy N.A., Md S., Kesharwani P. (2022). Recent Progress of RGD Modified Liposomes as Multistage Rocket Against Cancer. Front. Pharmacol..

[21] Javid H., Oryani M.A., Rezagholinejad N., Hashemzad A., Karimi-Shahri M.J. (2024). Unlocking the Potential pf RGD-Conjugated Gold Nanoparticles: A New Frontier in Targeted Cancer Therapy, Imaging, and Metastasis Inhibition. Mater. Chem. B.

[22] Lorenzoni S., Rodríguez-Nogales C., Blanco-Prieto M.J. (2025). Targeting Tumor Microenvironment with RGD-Functionalized Nanoparticles for Precision Cancer therapy. Cancer Lett..

[23] Yin L., Li X., Wang R., Zeng Y., Zheng Z., Xie T. (2023). Recent Research Progress of RGD Peptide–Modified Nanodrug Delivery Systems in Tumor Therapy. Int. J. Pept. Res. Ther..

[24] Javid H., Oryani M.A., Rezagholinejad N., Esparham A., Tajaldini M., Karimi-Shahri M. (2024). RGD peptide in cancer targeting: Benefits, challenges, solutions, and possible integrin-RGD interactions. Cancer Med..

[25] Xiong X.-B., Ma Z., Lai R., Lavasanifar A. (2010). The Therapeutic Response to Multifunctional Polymeric Nano-Conjugates in the Targeted Cellular and Subcellular Delivery of Doxorubicin. Biomaterials.

[26] Zhen Z., Tang W., Chen H., Lin X., Todd T., Wang G., Cowger T., Chen X., Xie J. (2013). RGD-Modified Apoferritin Nanoparticles for Efficient Drug Delivery to Tumors. ACS Nano.

[27] Anders J., Cortez A.G., Yu J., Majumdar S., Bhise A., Hobbs R.F., Nedrwo J.R. (2024). Evaluation of Targeting αVβ3 in Breast Cancers Using RGD Peptide-Based Agents. Nuclear Med. Biol..

[28] Sujin K., Lee S., Park S. (2020). iRGD Peptide as a Tumor-Penetrating Enhancer for Tumor-Targeted Drug Delivery. Polymers.

[29] Wang C., Li N., Li Y., Hou S., Zhang W., Meng Z., Wang S., Jia Q., Tan J., Wang R. (2022). Engineering a HEK-293T exosome-based delivery platform for efficient tumor-targeting chemotherapy/internal irradiation combination therapy. J. Nanobiotech..

[30] Meichen Z., Xu H. (2023). Peptide-Assembled Nanoparticles Targeting Tumor Cells and Tumor Microenvironment for Cancer Therapy. Front. Chem..

[31] Timur S.S., Yöyen-Ermiş D., Esendağlı G., Yonat S., Horzum U., Esendağlı G., Gürsoy R.N. (2019). Efficacy of a novel LyP-1-containing self-microemulsifying drug delivery system (SMEDDS) for active targeting to breast cancer. Eur. J. Pharm. Biopharm..

[32] Zhong Z., Cai L., Li C. (2020). Characterization and targeting ability evaluation of cell-penetrating peptide LyP-1 modified alginate-based nanoparticles. RSC Adv..

[33] Adeyemi S.A., Choonara Y.A. (2022). In Vitro and In Vivo Evaluation of a Cyclic LyP-1-Modified Nanosystem for Targeted Endostatin Delivery in a KYSE-30 Cell Xenograft Athymic Nude Mice Model. Pharmaceuticals.

[34] Guo G., Yu X., Jun C., Yang F. (2009). LyP-1-conjugated nanoparticles for targeting drug delivery to lymphatic metastatic tumors. Int. J. Pharm..

[35] Ma Y., Li R., Dong Y., You C., Huang S., Li X., Wang F., Zhang Y. (2021). tLyP-1 Peptide Functionalized Human H Chain Ferritin for Targeted Delivery of Paclitaxel. Int. J. Nanomed..

[36] Isha G., Thakur A., Zhang K., Thakur S., Hu X., Xu Z., Kumar G., Jaganathan R., Iyaswamy A., Li M. (2024). Peptide-Conjugated Vascular Endothelial Extracellular Vesicles Encapsulating Vinorelbine for Lung Cancer Targeted Therapeutics. Nanomaterials.

[37] Liu X., Cao Z., Liu N., Gao G., Du M., Wang Y., Cheng B., Zhu M., Jia B., Pan L. (2022). Kill two birds with one stone: Engineered exosome-mediated delivery of cholesterol modified YY1-siRNA enhances chemoradiotherapy sensitivity of glioblastoma. Front. Pharmacol..

[38] Cheng H.-Y., Hsieh C.-H., Lin P.-H., Chen Y.-T., Hsu D.S.-S., Tai S.-K., Chu P.-Y., Yang M.-H. (2022). Snail-regulated exosomal microRNA-21 suppresses NLRP3 inflammasome activity to enhance cisplatin resistance. J. Immunother. Cancer.

[39] Borrego-Soto G., Ortiz-López R., Rojas-Martínez A. (2015). Ionizing radiation-induced DNA injury and damage detection in patients with breast cancer. Genet. Mol. Biol..

[40] Eychenne R., Bouvry C., Bourgeois M., Loyer P., Benoist E., Lepareur N. (2020). Overview of Radiolabeled Somatostatin Analogs for Cancer Imaging and Therapy. Molecules.

[41] Trautwein N.F., Schwenck J., Jacoby J., Reischl G., Fiz F., Zender L., Dittmann H., Hinterleitner M., la Fougère C. (2023). Long-term prognostic factors for PRRT in neuroendocrine tumors. Front. Med..

[42] Hofland J., Brabander T., Verburg F.A., Feelders R.A., de Herder W.W. (2022). Peptide Receptor Radionuclide Therapy. J. Clin. End. Metab..

[43] Strosberg J.R., Al-Toubah T., El-Haddad G., Lagunes D.R., Bodei L. (2024). Sequencing of Somatostatin-Receptor-Based Therapies in Neuroendocrine Tumor Patients. J. Nucl. Med..

[44] Baum R.P., Schuchardt C., Singh A., Chantadisai M., Robiller F.C., Zhang J., Mueller D., Eismant A., Almaguel F., Zboralski D. (2022). Feasibility, Biodistribution, and Preliminary Dosimetry in Peptide-Targeted Radionuclide Therapy of Diverse Adenocarcinomas Using ^177^Lu-FAP-2286: First-in-Humans Results. J. Nucl. Med..

[45] Patell K., Kurian M., Garcia J.A., Mendiratta P., Barata P.C., Jia A.Y., Spratt D.E., Brown J.R. (2023). Lutetium-177 PSMA for the treatment of metastatic castrate resistant prostate cancer: A systematic review. Expert Rev. Anticancer Ther..

[46] Entezari P., Gabr A., Salem R., Lewandowski R.J. (2022). Yttrium-90 for colorectal liver metastasis—The promising role of radiation segmentectomy as an alternative local cure. Int. J. Hyperth..

[47] Otte A., Mueller-Brand J., Dellas S., Nitzsche E.U., Herrmann R., Maecke H.R. (1998). Yttrium-90 labeled somatostatin-analogue for cancer treatment. Lancet.

[48] Otte A., Herrmann R., Heppeler A., Behe M., Jermann E., Powell P., Maecke H.R., Muller J. (1999). Yttrium-90 DOTATOC: First clinical results. Eur. J. Nucl. Med..

[49] Vinjamuri S., Gilbert T.M., Banks M., McKane G., Maltby P., Poston G., Weissman H., Palmer D.H., Vora J., Pritchard D.M. (2013). Peptide Receptor Radionuclide Therapy With 90Y-DOTATATE/90Y-DOTATOC in Patients with Progressive Metastatic Neuroendocrine Tumours: Assessment of Response, Survival and Toxicity. Br. J. Cancer.

[50] Virgolini I., Britton K., Buscombe J., Moncayo R., Paganelli G., Riva P. (2002). In- and Y-DOTA-lanreotide: Results and implications of the MAURITIUS trial. Semin. Nucl. Med..

[51] Strosberg J., El-Haddad G., Wolin E., Hendifar A., Yao J., Chasen B., Mittra E., Kunz P.L., Kulke M.H., Jacene H. (2017). NETTER-1 Trial Investigators. Phase 3 Trial of 177Lu-Dotatate for Midgut Neuroendocrine Tumors. N. Engl. J. Med..

[52] Hoogenkamp D.S., de Wit-van der Veen L.J., Huizing D.M.V., Tesselaar M.E.T., van Leeuwaarde R.S., Stokkel M.P.M., Lam M.G.E.H., Braat A.J.A.T. (2024). Advances in Radionuclide Therapies for Patients with Neuro-endocrine Tumors. Curr. Oncol. Rep..

[53] Merola E., Grana C.M. (2023). Peptide Receptor Radionuclide Therapy (PRRT): Innovations and Improvements. Cancers.

[54] Alas M., Saghaeidehkordi A., Kaur K. (2021). Peptide-Drug Conjugates with Different Linkers for Cancer Therapy. J. Med. Chem..

[55] Cooper B.M., Iegre J., O’Donovan D.H., Halvarsson M.O., Spring D.R. (2021). Peptides as a platform for targeted therapeutics for cancer: Peptide–drug conjugates (PDCs). Chem. Soc. Rev..

[56] Balogh B., Ivánczi M., Nizami B., Beke-Somfai T., Mándity I.M. (2021). ConjuPepDB: A database of peptide-drug conjugates. Nucleic Acids Res..

[57] Tripodi A.A.P., Tóth S., Enyedi K.N., Scholosser G., Szakács G., Mező G. (2018). Development of novel cyclic NGR peptide–daunomycin conjugates with dual targeting property. J. Org. Chem..

[58] Zhu L., Ding Z., Li X., Wei H., Chen Y. (2020). Research Progress of Radiolabeled Asn-GlyArg (NGR) Peptides for Imaging and Therapy. Mol. Imaging.

[59] Ziaei E., de Paiva I.M., Yao S.-J., Sarrami N., Mehinrad P., Lai J., Lavasanifar A., Kaur K. (2023). Peptide-Drug Conjugate Targeting Keratin 1 Inhibits Triple-Negative Breast Cancer in Mice. Mol. Pharmacol..

[60] Jiang K., Song X., Yang L., Li L., Wan Z., Sun X., Gong T., Lin Q., Zhang Z. (2018). Enhanced antitumor and anti-metastasis efficacy against aggressive breast cancer with a fibronectin-targeting liposomal doxorubicin. J. Control. Release.

[61] Delavari B., Bigdeli B., Khazeni S., Varamini P. (2025). Nanodiamond-Protein hybrid Nanoparticles: LHRH receptor targeted and co-delivery of doxorubicin and dasatinib for triple negative breast cancer therapy. Int. J. Pharm..

[62] Emons G., Gorchev G., Harter P., Wimberger P., Stähle A., Hanker L., Hilpert F., Beckmann M.W., Dall P., Gründker C. (2014). Efficacy and safety of AEZS-108 (LHRH agonist linked to doxorubicin) in women with advanced or recurrent endometrial cancer expressing LHRH receptors: A multicenter phase 2 trial (AGO-GYN5). Int. J. Gynecol. Cancer.

[63] https://www.clinicaltrials.gov/study/NCT01767155?tab=results#outcome-measures.

[64] Xie M.-H., Fu Z.-L., Hua A.-L., Zhou J.-F., Chen Q., Li J.-B., Yao S., Cai X.-J., Ge M., Zhou L. (2022). A new core-shell-type nanoparticle loaded with paclitaxel/norcantharidin and modified with APRPG enhances anti-tumor effects in hepatocellular carcinoma. Front. Oncol..

[65] Fu C., Yu L., Miao Y., Liu X., Yu Z., Wei M. (2023). Peptide-drug conjugates (PDCs): A novel trend of research and development on targeted therapy, hype or hope?. Acta Pharm. Sin. B.

[66] Coburn F., Nsereko Y., Armstrong A., Musaimi O.A. (2025). Peptide inhibitors: Breaking cancer code. Eur. J. Med. Chem..

[67] Wintgens J.P., Wichert S.P., Popovic L., Rossner M.J., Wehr M.C. (2019). Monitoring activities of receptor tyrosine kinases using a universal adapter in genetically encoded split TEV assays. Cell Mol. Life Sci..

[68] Zhu C., Wei Y., Wei X. (2019). AXL receptor tyrosine kinase as a promising anti-cancer approach: Functions, molecular mechanisms and clinical applications. Mol. Cancer.

[69] Amato J.G., Erinn B.R., Jennifer R.C., Douglas J., Mihalis K., Katherine F., Yu M., Susan H. (2018). Modified AXL Peptides and Their Use in Inhibition of AXL Signaling in Anti-Metastatic Therapy. https://patents.google.com/patent/HK1256071B/en.

[70] Amato J.G., Erinn B.R., Jennifer R.C., Douglas J., Mihalis K., Katherine F., Yu M., Susan H. (2013). Modified AXL Peptides and Their Use in Inhibition of AXL Signaling in Anti-Metastatic Therapy. https://patents.google.com/patent/US9822347B2/en#:~:text=translated%20from.%20Compositions%20and%20methods%20are%20provided,MER%20or%20Tyro3%20and%20its%20ligand%20GAS6.

[71] William A.D., Flanagan J.U. (2021). Inhibitors of Discoidin Domain Receptor (DDR) Kinases for Cancer and Inflammation. Biomolecules.

[72] Kim D., Yeom J.-H., Lee B., Lee K., Bae J., Rhee S. (2015). Inhibition of discoidin domain receptor 2-mediated lung cancer cells progression by gold nanoparticle-aptamer-assisted delivery of peptides containing transmembrane-juxtamembrane 1/2 domain. Biochem. Biophys. Res. Comm..

[73] Borza C.M., Bolas G., Zhang X., Browning Monroe M.B., Zhang M.Z., Meiler J., Skwark M.J., Harris R.C., Lapierre L.A., Goldenring J.R. (2022). The Collagen Receptor Discoidin Domain Receptor 1b Enhances Integrin β1-Mediated Cell Migration by Interacting with Talin and Promoting Rac1 Activation. Front. Cell Dev. Biol..

[74] Carafoli F., Bihan D., Stathopoulos S., Konitsiotis A.D., Kvansakul M., Farndale R.W., Leitinger B., Hohenester E. (2009). Crystallographic Insight into Collagen Recognition by Discoidin Domain Receptor 2. Structure.

[75] Rosselot C., Li Y., Wang P., Alvarsson A., Beliard K., Lu G., Kang R., Li R., Liu H., Gillespie V. (2024). Harmine and exendin-4 combination therapy safely expands human β cell mass in vivo in a mouse xenograft system. Sci. Transl. Med..

[76] Mehta R.K., Shukla S., Ramanand S.G., Somnay V., Bridges A.J., Lawrence T.S., Nyati M.K. (2021). Disruptin, a cell-penetrating peptide degrader of EGFR: Cell-Penetrating Peptide in Cancer Therapy. Transl. Oncol..

[77] Ahsan A., Ramanand S.G., Bergin I.L., Zhao L., Whitehead C.E., Rehemtulla A., Ray D., Pratt W.B., Lawrence T.S., Nyati M.K. (2014). Efficacy of an EGFR-specific peptide against EGFR-dependent cancer cell lines and tumor xenografts. Neoplasia.

[78] Zhou Z., Zhao C., Wang L., Cao X., Li J., Huang R., Lao Q., Yu H., Li Y., Du H. (2015). A VEGFR1 antagonistic peptide inhibits tumor growth and metastasis through VEGFR1-PI3K-AKT signaling pathway inhibition. Am. J. Cancer Res..

[79] Farzaneh Behelgardi M., Zahri S., Mashayekhi F., Mansouri K., Asghari S.M. (2018). A peptide mimicking the binding sites of VEGF-A and VEGF-B inhibits VEGFR-1/-2 driven angiogenesis, tumor growth and metastasis. Sci. Rep..

[80] Lee J.S., Tocheny C.E., Shaw L.M. (2022). The Insulin-like Growth Factor Signaling Pathway in Breast Cancer: An Elusive Therapeutic Target. Life.

[81] Iams W.T., Lovly C.M. (2015). Molecular Pathways: Clinical Applications and Future Direction of Insulin-like Growth Factor-1 Receptor Pathway Blockade. Clin. Cancer Res..

[82] Mohamad N.-V., Ima-Nirwana S., Chin K.-Y. (2021). The Skeletal Effects of Gonadotropin-Releasing Hormone Antagonists: A Concise Review. Endocr. Metab. Immune Disord. Drug Targets.

[83] Connolly R.M., Carducci M.A., Antonarakis E.S. (2012). Use of androgen deprivation therapy in prostate cancer: Indications and prevalence. Asian J. Androl..

[84] (2012). Gonadotropin Releasing Hormone (GnRH) Analogues. LiverTox: Clinical and Research Information on Drug-Induced Liver Injury.

[85] Zhao J., Chen J., Sun G., Shen P., Zeng H. (2024). Luteinizing hormone-releasing hormone receptor agonists and antagonists in prostate cancer: Effects on long-term survival and combined therapy with next-generation hormonal agents. Cancer Biol. Med..

[86] Tombal B., Collin S., Morgans A.K., Hunsche E., Brown B., Zhu E., Bossi A., Shore N. (2023). Impact of Relugolix Versus Leuprolide on the Quality of Life of Men with Advanced Prostate Cancer: Results from the Phase 3 HERO Study. Eur. Urol..

[87] Perlmutter M.A., Lepor H. (2007). Androgen deprivation therapy in the treatment of advanced prostate cancer. Rev. Urol..

[88] Patel H.K., Bihani T. (2018). Selective estrogen receptor modulators (SERMs) and selective estrogen receptor degraders (SERDs) in cancer treatment. Pharmacol. Ther..

[89] Bennett J.A., Mesfin F.B., Andersen T.T., Gierthy J.F., Jacobson H.I. (2002). A peptide derived from α-fetoprotein prevents the growth of estrogen-dependent human breast cancers sensitive and resistant to tamoxifen. Proc. Natl. Acad. Sci. USA.

[90] Speltz T.E., Danes J.M., Stender J.D., Frasor J., Moore T.W. (2018). A Cell-Permeable Stapled Peptide Inhibitor of the Estrogen Receptor/Coactivator Interaction. ACS Chem. Biol..

[91] Joufrre B., Acramel A., Belnou M., Santolla M.F., Talia M., Lappano R., Nemati F., Decaudin D., Khemtemourian L., Lui W.-Q. (2023). Identification of a human estrogen receptor α tetrapeptidic fragment with dual antiproliferative and anti-nociceptive action. Sci. Rep..

[92] Lappano R., Mallet C., Rizzuti B., Grande F., Galli G.R., Byrne C., Broutin I., Boudieu L., Eschalier A., Jacquot Y. (2019). The Peptide ERα17p Is a GPER Inverse Agonist that Exerts Antiproliferative Effects in Breast Cancer Cells. Cells.

[93] Wander S.A., Cohen O., Gong X., Johnson G.N., Buendia-Buendia J.E., Lloyd M.R., Kim D., Luo F., Mao P., Helvie K. (2020). The Genomic Landscape of Intrinsic and Acquired Resistance to Cyclin-Dependent Kinase 4/6 Inhibitors in Patients with Hormone Receptor-Positive Metastatic Breast Cancer. Cancer Discov..

[94] Hamza S., Garanina E.E., Alsaadi M., Khaiboullina S.F., Tezcan G. (2023). Blocking the Hormone Receptors Modulates NLRP3 in LPS-Primed Breast Cancer Cells. Int. J. Mol. Sci..

[95] Nadal-Bufí F., Chan L.Y., Mohammad H.H., Mason J.M., Salomon C., Lai A., Thompson E.W., Craik D.J., Kaas Q., Henriques S.T. (2022). Peptide-based LDH5 inhibitors enter cancer cells and impair proliferation. Cell Mol. Life Sci..

[96] Yang N., Liang Y., Yang P., Jiang L. (2022). Flurbiprofen inhibits cell proliferation in thyroid cancer through interrupting HIP1R-induced endocytosis of PTN. Eur. J. Med. Res..

[97] Wang H., Yao H., Li C., Shi H., Lan J., Li Z., Zhang Y., Liang L., Fang J.-Y., Xu J. (2019). HIP1R targets PD-L1 to lysosomal degradation to alter T cell-mediated cytotoxicity. Nat. Chem. Biol..

[98] Chen H., Zhan M., Liu J., Liu Z., Shen M., Yang F., Kang Y., Yin F., Li Z. (2022). Structure-Based Design, Optimization, and Evaluation of Potent Stabilized Peptide Inhibitors Disrupting MTDH and SND1 Interaction. J. Med. Chem..

[99] Chen H., Zhan M., Zhang Y., Liu J., Wang R., An Y., Gao Z., Jiang L., Xing Y., Kang Y. (2023). Intracellular Delivery of Stabilized Peptide Blocking MTDH-SND1 Interaction for Breast Cancer Suppression. JACS Au.

[100] Zhou H., Zhou W., Zhou B., Liu L., Chern T.R., Chinnaswamy K., Lu J., Bernard D., Yang C.Y., Li S. (2018). High-Affinity Peptidomimetic Inhibitors of the DCN1-UBC12 Protein-Protein Interaction. J. Med. Chem..

[101] Yu X., Li D., Kottur J., Shen Y., Kim S.H., Park K.-S., Tsai Y.-H., Gong W., Wng J., Suzuki K. (2021). A selective WDR5 degrader inhibits acute myeloid leukemia in patient-derived mouse models. Sci. Transl. Med..

[102] Luan X., Wu Y., Shen Y.-W., Zhang H., Zhou Y.-D., Chen H.-Z., Nagle D.G., Zhang W.-D. (2021). Cytotoxic and antitumor peptides as novel chemotherapeutics. Nat. Prod. Rep..

[103] Kourie J.I., Shorthouse A.A. (2000). Properties of cytotoxic peptide-formed ion channels. Am. J. Physiol. Cell Physiol..

[104] Haque S., Hussain A., Joshi H., Sharma U., Sharma B., Aggarwal D., Rani I., Ramniwas S., Gupta M., Tuli H.S. (2023). Melittin: A possible regulator of cancer proliferation in preclinical cell culture and animal models. J. Cancer Res. Clin. Oncol..

[105] Pandey P., Khan F., Khan M.A., Kumar R., Upadhyay T.K. (2023). An Updated Review Summarizing the Anticancer Efficacy of Melittin from Bee Venom in Several Models of Human Cancers. Nutrients.

[106] Duffy C., Sorolla A., Wang E., Golden E., Woodward E., Davern K., Ho D., Johnstone E., Pfleger K., Redfern A. (2020). Honeybee venom and melittin suppress growth factor receptor activation in HER2-enriched and triple-negative breast cancer. NPJ Precis. Oncol..

[107] Zhang H., Zhao B., Huang C., Meng X.-M., Bian E.-B., Li J. (2014). Melittin Restores PTEN Expression by Down-Regulating HDAC2 in Human Hepatocelluar Carcinoma HepG2 Cells. PLoS ONE.

[108] Mao J., Liu S., Ai M., Wang Z., Wang D., Li X., Hu K., Gao X., Yang Y. (2017). A novel melittin nano-liposome exerted excellent anti-hepatocellular carcinoma efficacy with better biological safety. J. Hematol. Oncol..

[109] Ye H., Lei M. (2025). 124P Melittin inhibits the growth of hepatocellular carcinoma Huh7 cells by downregulating LARS2 and ZNF19. Eur. Soc. Med. Oncol..

[110] Zhu Z., Chen W.-Q., Zhang S.-Q., Bai J.-X., Lau C.-L., Sze S.C.-W., Yung K.K.-L., Ko J.K.-S. (2022). The human cathelicidin peptide LL-37 inhibits pancreatic cancer growth by suppressing autophagy and reprogramming of the tumor immune microenvironment. Front. Pharmacol..

[111] Chen X., Zou X., Qi G., Tang Y., Guo Y., Si J., Liang L. (2018). Roles and Mechanisms of Human Cathelicidin LL-37 in Cancer. Cell. Physiol. Biochem..

[112] Anghel R., Jitaru D., Bădescu L., Bădescu M., Ciocoiu M. (2013). The cytotoxic effect of magainin II on the MDA-MB-231 and M14K tumour cell lines. BioMed Res. Int..

[113] Lehmann J., Retz M., Sidhu S.S., Suttmann H., Sell M., Paulsen F., Harder J., Unteregger G. (2006). Antitumor Activity of the Antimicrobial Peptide Magainin II against Bladder Cancer Cell Lines. Eur. Urol..

[114] Liu S., Yang H., Cheng J., Lu X. (2013). Penetratin-Mediated Delivery Enhances the Antitumor Activity of the Cationic Antimicrobial Peptide Magainin II. Cancer Biother. Radiopharm..

[115] Baker M.A., Maloy W.L., Zasloff M., Jacob L.S. (1993). Anticancer efficacy of Magainin2 and analogue peptides. Cancer Res..

[116] Pandurangi R., Karwa A., Sagaram U.S., Henzler-Wildman K., Shah D. (2023). Medicago Sativa Defensin1 as a tumor sensitizer for improving chemotherapy: Translation from antifungal agent to a potential anti-cancer agent. Front. Oncol..

[117] Amaral V.S.G.D., Santos S.A.C.S., de Andrade P.C., Nowatzki J., Júnior N.S., de Medeiros L.N., Gitirana L.B., Pascutti P.G., Almeida V.H., Monteiro R.Q. (2020). *Pisum sativum* Defensin 1 Eradicates Mouse Metastatic Lung Nodules from B16F10 Melanoma Cells. Int. J. Mol. Sci..

[118] Baxter A., Poon I., Hulett M. (2017). The plant defensin NaD1 induces tumor cell death via a non-apoptotic, membranolytic process. Cell Death Discov..

[119] Pandurangi R., Sekar T., Paulmurugan R. (2024). Restoration of the Lost Human Beta Defensin-1 Protein in Cancer as a Strategy to Improve the Efficacy of Chemotherapy. J. Med. Chem..

[120] Sun C.Q., Arnold R.S., Hsieh C.L., Dorin J.R., Lian F., Li Z., Petros J.A. (2019). Discovery and mechanisms of host defense to oncogenesis: Targeting the β-defensin-1 peptide as a natural tumor inhibitor. Cancer Biol. Ther..

[121] Adyns L., Proost P., Struyf S. (2023). Role of defensins in tumor biology. Int. J. Mol. Sci..

[122] Müller C.A., Markovic-Lipkovski J., Klatt T., Gamper J., Schwarz G., Beck H., Deeg M., Kalbacher H., Widmann S., Wessels J.T. (2002). Human alpha-defensins HNPs-1, -2, and -3 in renal cell carcinoma: Influences on tumor cell proliferation. Am. J. Pathol..

[123] Semple F., Dorin J.R. (2012). β-Defensins: Multifunctional modulators of infection, inflammation and more?. J. Innate Immun..

[124] Nagib M., Sayed A.M., Korany A.H., Abdelkader K., Shari F.H., Mackay W.G., Rateb M.E. (2025). Human Defensins: Structure, Function, and Potential as Therapeutic Antimicrobial Agents with Highlights Against SARS CoV-2. Probiotics Antimicrob. Proteins.

[125] Zhang D., Wan L., Zhang J., Liu C., Sun H. (2015). Effect of BMAP-28 on human thyroid cancer TT cells is mediated by inducing apoptosis. Oncol. Lett..

[126] Wu M.-C., Wang E.Y., Lai T.W. (2023). TAT peptide at treatment-level concentrations crossed brain endothelial cell monolayer independent of receptor-mediated endocytosis or peptide-inflicted disruption. PLoS ONE.

[127] He Y., Lu H., Zhao Y. (2022). Development of an autophagy activator from Class III PI3K complexes, Tat-BECN1 peptide: Mechanisms and applications. Front. Cell Dev. Biol..

[128] di Polidoro A.C., Cafarchio A., Vecchione D., Donato P., de Nola F., Torino E. (2022). Revealing Angiopep-2/LRP1 Molecular Interaction Optimal Delivery to Glioblastoma (GBM). Molecules.

[129] Kumthekar P., Tang S.-C., Brenner A.J., Kesari S., Piccioni D.E., Anders C., Carrillo J., Chalasani P., Kabos P., Puhalla S. (2020). ANG1005, a Brain-Penetrating Peptide-Drug Conjugate, Shows Activity in Patients with Breast Cancer with Leptomeningeal Carcinomatosis and Recurrent Brain Metastases. Clin. Cancer Res..

[130] Dmello C., Brenner A., Piccioni D., Wen P.Y., Drappatz J., Mrugala M., Lewis L.D., Schiff D., Fadul C.E., Chamberlain M. (2024). Phase II trial of blood-brain barrier permeable peptide-paclitaxel conjugate ANG1005 in patients with recurrent high-grade glioma. Neuro-Oncol. Adv..

[131] Lei Y., Liu J., Bai Y., Zheng C., Wang D. (2025). Peptides as Versatile Regulators in Cancer Immunotherapy: Recent Advances, Challenges, and Future Prospects. Pharmaceutics.

[132] Anilkumar A.S., Thomas S.M., Veerabathiran R. (2025). Next-generation cancer vaccines: Targeting cryptic and non-canonical antigens for precision immunotherapy. Explor. Target. Antitumor Ther..

[133] Liu D., Liu L., Li X., Wang S., Wu G., Che X. (2024). Advancements and Challenges in Peptide-Based Cancer Vaccination: A Multidisciplinary Perspective. Vaccines.

[134] Hamley I.W. (2022). Peptides for Vaccine Development. ACS Appl. Bio Mater..

[135] Gupta M., Whi A., Sharma P., Nagpal P., Raina N., Kaurav M., Bhattacharya J., Rodrigues Oliveuram S.M., Dolma K.G., Paul A.L. (2022). Recent Advances in Cancer Vaccines: Challenges, Achievements, and Futuristic Prospects. Vaccines.

[136] Liu W., Tang H., Li L., Wang X., Yu Z., Li J. (2021). Peptide-based therapeutic cancer vaccine: Current trends in clinical application. Cell Prolif..

[137] Abd-Aziz N., Poh C.L. (2022). Development of Peptide-Based Vaccines for Cancer. J. Oncol..

[138] Hongo F., Ueda T., Takasha N., Tamada S., Nakatani T., Miki T., Ukimura O. (2023). Phase I/II study of multipeptide cancer vaccine IMA901 after single-dose cyclophosphamide in Japanese patients with advanced renal cell cancer with long-term follow up. Int. J. Urol..

[139] O’Shea A.E., Clifton G.T., Qiao N., Heckman-Stoddard B.M., Wojtowicz M., Dimond E., Bedrosian I., Weber D., Garber J.E., Husband A. (2023). Phase II Trial of Nelipepimut-S Peptide Vaccine in Women with Ductal Carcinoma In Situ. Cancer Prev. Res..

[140] You Z., Zhou W., Weng J., Feng H., Liang P., Li Y., Shi F. (2021). Application of HER2 peptide vaccines in patients with breast cancer: A systematic review and meta-analysis. Cancer Cell Int..

[141] Mittendorf E.A., Ardavanis A., Litton J.K., Shumway N.M., Hale D.F., Murray J.L., Perez S.A., Ponniah S., Baxevanis C.N., Papamichail M. (2016). Primary analysis of a prospective, randomized, single-blinded phase II trial evaluating the HER2 peptide GP2 vaccine in breast cancer patients to prevent recurrence. Oncotarget.

[142] Schneble E.J., Perez S.A., Murray J.L., Berry J.S., Trappey A.F., Vreeland T.J., Hale D.F., Mittendorf E.A. (2014). Primary analysis of the prospective, randomized, phase II trial of GP2+GM-CSF vaccine versus GM-CSF alone administered in the adjuvant setting to high-risk breast cancer patients. J. Clin. Oncol..

[143] Clifton G., Litton J.K., Arrington K., Ponniah S., Ibrahim N.K., Gall V., Alatrash G., Peoples G.E., Mittendorf E.A. (2017). Results of a Phase Ib Trial of Combination Immunotherapy with a CD8+ T Cell Eliciting Vaccine and Trastuzumab in Breast Cancer Patients. Ann. Surg. Oncol..

[144] Brown T.A., Mittendorf E.A., Hale D.F., Myers J.W., Peace K.M., Jackson D.O., Greene J.M., Vreeland T.J., Clifton G.T., Ardavanis A. (2020). Prospective, randomized, single-blinded, multi-center phase II trial of two HER2 peptide vaccines, GP2 and AE37, in breast cancer patients to prevent recurrence. Breast Cancer Res. Treat..

[145] CenterWatch Study. https://www.centerwatch.com/clinical-trials/listings/NCT05232916/phase-3-study-to-evaluate-the-efficacy-and-safety-of-her2-neu-peptide-glsi-100-gp2-gm-csf-in-her2neu-positive-subjects?utm=.

[146] Kim Y., Lee D., Go C., Yang J., Kang D., Kang J.S. (2021). GV1001 interacts with androgen receptor to inhibit prostate cell proliferation in benign prostatic hyperplasia by regulating expression of molecules related to epithelial-mesenchymal transition. Aging.

[147] Brunsvig P.F., Kyte J.A., Kersten C., Sundstrom S., Moller M., Nyakas M., Hansen G.L., Gaudernack G., Aamdal S. (2011). Telomerase Peptide Vaccination in NSCLC: A Phase II Trial in Stage III Patients Vaccinated after Chemoradiotherapy and an 8-Year Update on a Phase I/II Trial. Clin. Cancer Res..

[148] Negrini S., De Palma R., Filaci G. (2020). Anti-Cancer Immunotherapies Targeting Telomerase. Cancers.

[149] Kim J.H., Cho Y.R., Ahn E.K., Kim S., Han S., Kim S.J., Bae G.U., Oh J.S., Seo D.W. (2022). A novel telomerase-derived peptide GV1001-mediated inhibition of angiogenesis: Regulation of VEGF/VEGFR-2 signaling pathways. Transl. Oncol..

[150] Vahidi S., Zabeti Touchaei A. (2024). Telomerase-based vaccines: A promising frontier in cancer immunotherapy. Cancer Cell Int..

[151] Chen W., Shin K., Kim S., Shon W., Kim R.H., Park N., Kang M.K. (2018). hTERT peptide fragment GV1001 demonstrates radioprotective and antifibrotic effects through suppression of TGF-β signaling. Int. J. Mol. Med..

[152] Jo J.H., Kim Y.T., Choi H.S., Kim H.G., Lee H.S., Choi Y.W., Kim D.U., Lee K.H., Lim E.J., Han J.-H. (2024). Efficacy of GV1001 with gemcitabine/capecitabine in previously untreated patients with advanced pancreatic ductal adenocarcinoma having high serum eotaxin levels (KG4/2015): An open-label, randomised, Phase 3 trial. Br. J. Cancer.

[153] Middleton G., Silcocks P., Cox T., Valle J., Wadsley J., Propper D., Coxon F., Ross P., Madhusudan S., Roques T. (2014). Gemcitabine and capecitabine with or without telomerase peptide vaccine GV1001 in patients with locally advanced or metastatic pancreatic cancer (TeloVac): An open-label, randomised, phase 3 trial. Lancet Oncol..

[154] Kim J.Y., Yang D.W., Kim S., Choi J.G. (2023). Retrospective Analysis of the Clinical Characteristics of Patients with Breast Cancer Treated with Telomerase Peptide Immunotherapy Combined with Cytotoxic Chemotherapy. Breast Cancer.

[155] Wang S.J., Dougan S.K., Dougan M. (2023). Immune mechanisms of toxicity from checkpoint inhibitors. Trends Cancer.

[156] Cook S.L., Amin M.A., Bari S., Poonnen P.J., Khasraw M., Johnson M.O. (2024). Immune Checkpoint Inhibitors in Geriatric Oncology. Curr. Oncol. Rep..

[157] Nagasaki J., Ishino T., Togashi T. (2022). Mechanisms of resistance to immune checkpoint inhibitors. Cancer Sci..

[158] Wabitsch S., Tandon M., Ruf B., Zhang Q., McCallen J.D., McVey J.C., Ma C., Green B.L., Diggs L.P., Heinrich B. (2021). Anti-PD-1 in Combination with Trametinib Suppresses Tumor Growth and Improves Survival of Intrahepatic Cholangiocarcinoma in Mice. Cell. Mol. Gastroenterol. Hepatol..

[159] Liu H., Zhao Z., Zhang L., Li Y., Jain A., Barve A., Jun W., Liu Y., Fetse J., Cheng K. (2019). Discovery of low-molecular weight anti-PD-L1 peptides for cancer immunotherapy. J. Immunother. Cancer.

[160] Abbas A.B., Lin B., Liu C., Morshed A., Hu J., Xu H. (2019). Design and Synthesis of A PD-1 Binding Peptide and Evaluation of Its Anti-Tumor Activity. Int. J. Mol. Sci..

[161] Zhong T., Zhao C., Wang S., Tao D., Ma S., Shou C. (2020). The biologically functional identification of a novel TIM3-binding peptide P26 in vitro and in vivo. Cancer Chem. Pharmacol..

[162] Zhang X., Wang P., Shi G., Tang C., Xue H. (2024). AUNP-12 Near-Infrared Fluorescence Probes across NIR-I to NIR-II Enable *In Vivo* Detection of PD-1/PD-L1 Axis in the Tumor Microenvironment. Bioconjugate Chem..

[163] Podlesnykh S.V., Abramova K.E., Gordeeva A., Khlebnikov A.I. (2021). Peptide Blocking CTLA-4 and B7-1 Interaction. Molecules.

[164] Zheng B., Wang X., Guo M., Tzeng C.-M. (2025). Therapeutic Peptides: Recent Advances in Discovery, Synthesis, and Clinical Translation. Int. J. Mol. Sci..

[165] Yan W., Maki M.A., Mani V.R.M., Kumar P.V. (2025). Functional Peptides in Targeted Cancer Therapy: Mechanisms, Delivery Strategies, and Clinical Perspectives. Int. J. Pept. Res. Ther..

[166] Chavda V.P., Solanki H.K., Davidson M., Apostolopoulos V., Bojarska J. (2022). Peptide-Drug Conjugates: A New Hope for Cancer Management. Molecules.

[167] Teleanu R.I., Preda M.D., Niculescu A.G., Vladâcenco O., Radu C.I., Grumezescu A.M., Teleanu D.M. (2022). Current Strategies to Enhance Delivery of Drugs across the Blood-Brain Barrier. Pharmaceutics.

[168] Peng H., Wang J., Chen J., Peng Y., Wang X., Chen Y., Kaplan D.L., Wang Q. (2023). Challenges and opportunities in delivering oral peptides and proteins. Expert. Opin. Drug Deliv..

[169] https://www.genscript.com/recommended_peptide_purity.html#:~:text=Peptides%20with%20purity%20greater%20than,are%20excellent%20for%20quantitative%20analysis.

[170] Liu M., Svirskis D., Proft T., Loh J., Yin N., Li H., Zhou Y., Chen S., Song L., Chen G. (2025). Progress in peptide and protein therapeutics: Challenges and strategies. Acta Pharm. Sin. B.

[171] Pereira A.J., de Campos L.J., Xing H., Conda-Sheridan M. (2024). Peptide-based therapeutics: Challenges and solutions. Med. Chem. Res..

